# The logic of universalization guides moral judgment

**DOI:** 10.1073/pnas.2014505117

**Published:** 2020-10-02

**Authors:** Sydney Levine, Max Kleiman-Weiner, Laura Schulz, Joshua Tenenbaum, Fiery Cushman

**Affiliations:** ^a^Department of Psychology, Harvard University, Cambridge, MA 02138;; ^b^Department of Brain and Cognitive Sciences, Massachusetts Institute of Technology, Cambridge, MA 02139

**Keywords:** moral judgment, moral development, universalization

## Abstract

To explain why an action is wrong, we sometimes say, “What if everybody did that?” In other words, even if a single person’s behavior is harmless, that behavior may be wrong if it would be harmful once universalized. We formalize the process of universalization in a computational model, test its quantitative predictions in studies of human moral judgment, and distinguish it from alternative models. We show that adults spontaneously make moral judgments consistent with the logic of universalization, and report comparable patterns of judgment in children. We conclude that, alongside other well-characterized mechanisms of moral judgment, such as outcome-based and rule-based thinking, the logic of universalizing holds an important place in our moral minds.

## Significance

Humans have several different ways to decide whether an action is wrong: We might ask whether it causes harm or whether it breaks a rule. Moral psychology attempts to understand the mechanisms that underlie moral judgments. Inspired by theories of “universalization” in moral philosophy, we describe a mechanism that is complementary to existing approaches, demonstrate it in both adults and children, and formalize a precise account of its cognitive mechanisms. Specifically, we show that, when making judgments in novel circumstances, people adopt moral rules that would lead to better consequences if (hypothetically) universalized. Universalization may play a key role in allowing people to construct new moral rules when confronting social dilemmas such as voting and environmental stewardship.

Many people feel morally obligated to vote (1), recycle (2), and contribute to the public good in general (3, 4). Yet, current theories of moral psychology have trouble explaining why. We know that people sometimes judge actions according to the utilitarian principle of whether they help or harm others (5–9). But a single person’s decision to vote in a national election, for instance, almost certainly makes no difference. Other times, people judge actions according to the emotions they elicit—when judging, for instance, stabbing a person (5, 6), or french kissing your sibling (10). Voting and recycling, however, are rarely so arousing. Other times, people judge actions wrong when they violate clear social norms (11) or rules (9, 12–14). But to skip voting is legal and commonplace.

Why, then, does anybody consider it wrong to skip voting, or to withhold contributing to similar public goods? Ask, and sooner or later you’ll hear something like this: “Imagine what would happen if everybody did that!” This logic arises in everyday assessments of different social dilemmas. Why not pick flowers for your home from the nice bushes in the public park? Why not take all of the money from the change jar at the checkout counter and buy yourself a chocolate? Why not flush a paper napkin down the toilet at work? To any of these questions, a person might reasonably respond, “What if everybody did that?” Our goal is to understand what they mean, whether they really mean it, and what it means for current theories of moral psychology.

## Universalization

We call this mechanism for making moral judgments “universalization”: People decide whether it is morally permissible for a person to perform an action by asking what would happen if (hypothetically) everybody felt free to do the same. The better things would be expected to go, the more likely the action is judged permissible. The worse things would be expected to go, the less so.

Universalization differs from the dominant psychological models of moral judgment. According to utilitarian models, people ask “What would actually happen if I did that?” not “What would hypothetically happen if everyone did?” The process of universalization is grounded neither in automatic emotional responses nor in existing social norms and rules.

Rather, universalization generates new rules by considering their hypothetical consequences. This involves a distinct composition of elements essential to other theories of moral psychology. Specifically, universalization respects the joint constraints of utility (“What would happen…”), impartiality (“…if everybody…”), and rules (“followed this principle?”). It is a recurrent theme in philosophical theories ranging from Kant’s categorical imperative (15) to rule utilitarian theories (16–18). It also echoes agreement-based methods of collective moral decision-making, such as bargaining or negotiation (19, 20), and the associated philosophical tradition of contractualism (21–24). Like successful bargaining, universalization guides us toward impartial rules ensuring mutual benefit.

We do not propose—nor do our studies suggest—that universalization is the only mechanism of moral judgment, or the dominant one. Instead, we show that it is invoked in one particular and important kind of social dilemma, which we call “threshold problems.” We focus on these because they are highly diagnostic of universalization. In *Discussion*, we return to consider how often people rely on universalization, how it may apply beyond threshold problems, and how it relates to other well-established methods of moral judgment.

### Threshold Problems.

We define threshold problems by a basic structure: If only a few people do a particular action, nobody is harmed, but, when many people do it (i.e., more than the “threshold” number), everyone is harmed. Because universalizing asks what happens when everyone abides by the same principles, it renders distinctive moral judgments in these cases. For instance, consider a fishery where a new and more powerful fishing hook becomes available. If only one person uses the hook, then that person is better off, nobody else is worse off, and so overall utility increases. But, if lots of people use the new hook, the fishery will collapse. Thus, if everyone feels morally at liberty to use the hook (and a sufficient number are interested in doing so), overall utility will decrease. According to the logic of universalization, it is therefore wrong for even one person to start using the hook.

**Fig. 1.**
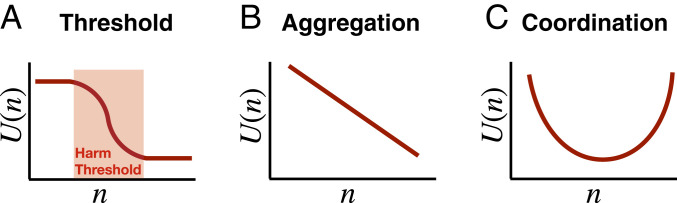
Three categories of social dilemmas and their utility functions. (*A*) Threshold problems (such as overfishing) possess a threshold structure where utility is high at small values of n, is low at high values of n, and decreases exclusively within an intermediate range. (*B*) Littering, on the other hand, is an aggregation problem, where each piece of litter adds the same negative utility to the outcome, and thus there is no delimited threshold range. (*C*) Another class of problems, coordination problems, have a high expected utility if everyone either decides to take the action or not take the action (such as driving on the left side of the road).

Fig. 1 defines the structure of a threshold problem in terms of the relationship between the number n of people who take the action and the resulting aggregate utility U(n). In threshold problems, there is a critical range of people acting for which U(n) decreases, but outside of which it does not. We call this range the *harm threshold*, because it is the threshold past which harm occurs. This contrasts with utility functions for other kinds of social dilemmas. For example, in aggregation problems, U(n) is strictly decreasing. An example of an aggregation problem is armed robbery: Each unique instance decreases total utility by roughly the same amount. In coordination problems, U(n) is maximized when everybody acts identically. Examples include driving on the right versus the left side of the road. Intuitively, universalization does not make sense in these cases: We would not explain why it is wrong for one person to rob another, or drive on the left side of the road, by asking, “What if everyone did that?”

The logic of universalization can explain why certain actions are wrong in aggregation and coordination problems, but so can other theories. For instance, psychological theories of utilitarian moral judgment can explain why unilateral defection is impermissible in aggregation and coordination problems, because a single person taking an action makes others worse off (i.e., U(1)<U(0)). But these alternative theories have trouble explaining why unilateral defection is impermissible in threshold problems, because a single person taking an action makes nobody worse off while bettering herself (i.e., U(1)≥U(0)). For instance, if one fisherman unilaterally begins to catch more fish with a new hook, he may be able to catch more fish without changing the size of anybody else’s catch. While his action does not actually reduce utility, it would hypothetically reduce total utility if universalized as an impartial rule. This is why the logic of universalization makes its most distinctive predictions in threshold problems.

### The Logic of Universalization.

The version of universalization we study here involves imagining the hypothetical consequences of everyone feeling at liberty to act a certain way.

Although the colloquial phrase, “What if everybody did that” implies that we universalize performing an action, this literal interpretation leads to absurd conclusions. It would be wrong, for instance, to be a dentist. After all, what if everyone became a dentist? (Clean teeth, but social collapse.)

We propose that people universalize not the action itself but the sense of moral liberty or moral constraint that attaches to it (25), better captured by the question “What if everyone felt free to do that?” (21). If everyone felt at liberty to skip voting, for instance, the civic outcome might be bad. But if everyone felt at liberty to be a dentist (as, presumably, we do), the outcome would be just fine: Liberty or not, most people are uninterested in dentistry.

Thus, we predicted that, in threshold problems, people would make moral judgments sensitive to the number of “interested parties” $n_{i}$—all those who would perform the action if they felt morally at liberty to do so. Specifically, we predicted that people would be more likely to judge an action wrong when the utility of all of the interested parties acting is worse than the utility of nobody acting, that is, $U\left(n_{i}\right)<U\left(0\right)$. This will happen, of course, if the number of interested parties exceeds the harm threshold. For example, suppose a fishery can sustain up to seven people using a new hook. If only three are interested, than the principle “use it if you want!” can be universalized with no harm. But, if 10 fishermen are interested, then universalizing the principle would cause harm, and therefore universalization predicts that it is impermissible for any of them to use it. Pursuing this logic, we test the prediction that moral judgments will be sensitive to the number of interested parties, in study 2a, and its interaction with the critical utility threshold, in study 2b and study 4.

A second key question is precisely what “utility” people are concerned with. Perhaps people are concerned with the personal utility of the actor. Or, perhaps they are concerned with the social utility of all interested parties. We test these and other possibilities in Study 3.

We are not the first to propose universalization as a mechanism for moral judgment. An analogous idea was the hallmark of the moral philosophy of Kant (15), which he called the “categorical imperative.” Similar ideas have been proposed in the normative theories of R. M. Hare (17), Marcus Singer (16), and others (26). Lawrence Kohlberg (27) suggested universalization as a psychological mechanism for making moral judgments. However, Kohlberg argued that this sophisticated form of reasoning emerged only in adults, and, typically, only after explicit philosophical training (28). We explore the developmental emergence of universalization in study 5.

Our work makes three main contributions. First, we state a formal model of universalization with sufficient precision to generate distinctive qualitative and quantitative predictions. Second, we show that many adults spontaneously use universalization to make moral judgments in threshold problems. Third, we provide evidence for universalization in childhood.

## Formalizing Universalization and Its Alternatives

We begin by defining an idealized model of universalization as applied to threshold problems, along with several alternative models of moral judgment. Formalization allows us to clearly organize each model’s competing qualitative predictions, and also to test their quantitative fit to experimental data.

### Universalization.

**Fig. 2.**
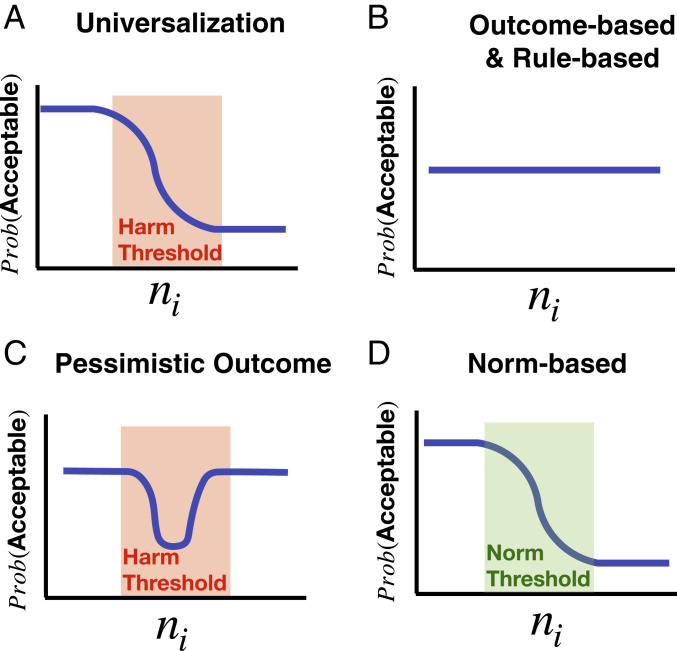
Predictions of several models of moral judgment. The graphs show the relationship between the number of interested parties $n_{i}$ and the probability of judging one person’s action morally acceptable, assuming none of the interested parties will actually act. (*A*) Universalization is sensitive to the number of interested parties and the harm threshold. (*B*) Simple outcome-based and rule-based models are not sensitive to the number of interested parties. (*C*) The pessimistic outcome model assumes that interested parties will act, and thus considers the action of one person to be least acceptable in the range of the harm threshold; here their action makes the greatest difference to outcomes. (*D*) A norm-based model is sensitive to the proportion of people who endorse a prohibitive norm. This may be approximated by the number of interested parties in some cases, as discussed in study 2, but this model is not sensitive to harm thresholds.

When universalizing, people compare the utilities of two hypothetical worlds in which different numbers of people (n) do the act in question: one in which n=0 because everybody feels morally constrained, and one in which $n=n_{i}$, the number of “interested parties”—i.e., those who would perform the action if they felt morally unconstrained. Following a common approach in models of choice (29, 30), we model moral judgment as a stochastic relationship of difference in utility between these hypothetical worlds, $U\left(0\right)-U\left(n_{i}\right)$, as given by the logistic (or “softmax”) function

$$P_{\text{Univ}}\left(\text{Acceptable}\right)=\frac{1}{1+e^{\tau \left(U\left(0\right)-U\left(n_{i}\right)\right)+\beta }},$$ [1]

where the “temperature” τ governs the strength of the effect of utility maximization on moral judgment, and the “bias” β governs whether people err on the side of acceptability or unacceptability judgments when the relevant utilities are approximately equal (Fig. 2*A*). This model uniquely predicts that moral acceptability is a function of both 1) the number of interested parties and 2) the utility function U(n) (that is, how utility changes as the number of people doing the action, n, increases). In threshold problems specifically, universalization predicts that moral judgment will change dramatically when $n_{i}$ exceeds the harm threshold on U.

### Outcome-Based.

Whereas the universalization model predicts that moral acceptability will be a function of the number of interested parties, $n_{i}$, standard outcome- or rule-based methods of moral judgment would not. A standard outcome-based model applied to our cases would depend on the difference in utility between the current actual number of people performing the action $n_{a}$ and the utility obtained if one more person did, that is, $U\left(n_{a}\right)-U\left(n_{a}+1\right)$. Again, we model moral judgment by applying a logistic function to this comparison:

$$P_{\text{Outcome}}\left(\text{Acceptable}\right)=\frac{1}{1+e^{\tau \left(U\left(n_{a}\right)-U\left(n_{a}+1\right)\right)+\beta }},$$ [2]

where τ and β have the same interpretation as above. Although this decision rule is sensitive to utility and makes use of the utility function U, it is not directly sensitive to the number of interested parties $n_{i}$ (Fig. 2*B*).

### Pessimistic Outcome.

We also consider a modified version of an outcome-based model that pessimistically assumes that anybody who is currently interested in performing some action will eventually actually do it. Thus, substituting $n_{a}=n_{i}$ into Eq. **2** yields

$$P_{\text{Pess. Out.}}\left(\text{Acceptable}\right)=\frac{1}{1+e^{\tau \left(U\left(n_{i}\right)-U\left(n_{i}+1\right)\right)+\beta }}.$$ [3]

Like universalization, this model is sensitive to the number of interested parties and to the harm threshold. But, it predicts a very different pattern of moral judgments (Fig. 2*C*). An action is judged wrong only when the number of interested parties falls within the range of the “harm threshold”; only here could adding one more actor plausibly influence outcomes. In other words, this model asks, “How likely am I to be the pivotal straw that breaks the camel’s back?” Below the harm threshold, adding an actor carries little risk of harm; above this zone, utility is doomed anyway, and additional actors make no difference. Moreover, in all of the experiments we conduct below, we make it clear that interested parties will not actually perform the action, violating the pessimistic assumption of this model. We ask participants whether they accept this premise, and exclude those who do not.

### Rule-Based.

A standard rule-based model would be sensitive to neither the number of interested parties nor the harm threshold. Rather, it predicts acceptability judgments as a function of the presence or absence of a rule,

$$P_{\text{Rule}}\left(\text{Acceptable}\right)=\left\{\begin{array}{cc} p\; & \text{if}\;\text{no}\;\text{rule}\\ 1-p\; & \text{if rule} \end{array}\right.,$$ [4]

where p governs the influence of rules on moral judgment (Fig. 2*B*).

### Norm-Based.

A third family of models (31) proposes that people judge an action wrong by considering how many other people judge it wrong—that is, whether there is a norm against it. Thus, it is not directly sensitive to the utility function U, but rather to the proportion of people subscribing to a prohibitive norm $n_{p}/n$,

$$P_{\text{Norm}}\left(\text{Acceptable}\right)=\frac{1}{1+e^{\tau \left(n_{p}/n\right)+\theta }},$$ [5]

where the “temperature” τ governs the influence of descriptive norms on moral judgment, and the “threshold” 0<θ<1 governs the threshold proportion of the population that must exhibit a norm in order for an agent to be more likely than not to also exhibit it (Fig. 2*D*). This model does not directly predict sensitivity to the number of interested parties, but, in study 2, we consider the possibility of an indirect relation where $n_{p}$ is approximated by $n_{i}$ (roughly, because interested parties who don’t act might be inferred to adhere to a moral norm).

### Experimental Approach.

Study 1 presents evidence that people explicitly endorse the logic of universalization in threshold cases. Study 2 rules out the alternative models based on the distinctive qualitative predictions each model makes. Study 2a rules out the rule, outcome, and pessimistic outcome models. Study 2b rules out the norms model. Study 3 differentiates between two versions of the universalization model. Study 4 compares idealized utility functions with subjective utility functions to establish a quantitative fit of the universalization model to the data. This study also looks at individual differences in patterns of moral judgment. Study 5 extends these methods to children 4 y to 11 y old.

## Study 1: Participants Endorse Universalization

We begin by testing whether people explicitly endorse the logic of universalization when presented with threshold problems. To do this, we presented participants with a variety of short descriptions of moral violations of different kinds, including but not limited to threshold problems. We then offered participants several different explanations for why those actions were morally wrong, and asked whether each was convincing. We predicted that participants would selectively endorse universalization for threshold problems.

We designed the violations to fall into four types: harm (e.g., hitting a person), fairness (e.g., not sharing resources), utility maximization (e.g., helping a small number of people when you could have helped many), and universalization (e.g., taking more fish than is sustainable). We presented participants with a menu of four moral explanations, each designed to match one of the categories of violations: 1) “because that harmed someone,” 2) “because that was unfair,” 3) “because that person could have helped more people,” and 4) “because if everyone did that, the outcome would be bad.” For each explanation, participants indicated whether or not it was a convincing reason for why that action was wrong; it was possible for a participant to judge multiple explanations convincing for each story—or none at all.

**Fig. 3.**
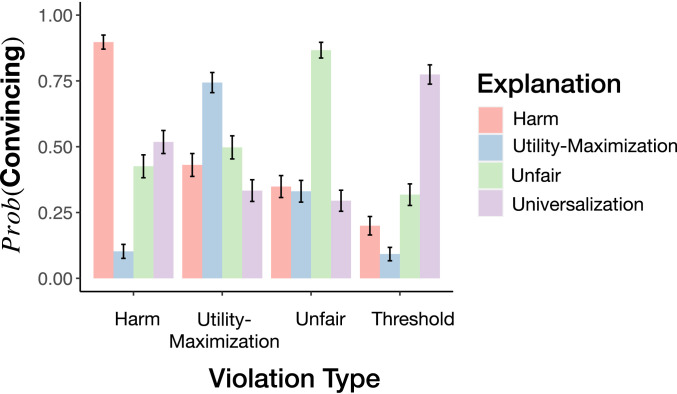
Study 1 results. Subjects endorse universalization as an explanation for threshold problems preferentially over harm-based, fairness-based, and utility-based explanations. Likewise, those explanations are selected for the appropriate moral violations. Error bars are SEM.

As predicted, participants strongly preferred universalization to explain why an individual action is wrong in a threshold problem (Fig. 3); 77% of responses to the threshold problems indicated that universalization was a good explanation of moral wrongness in that case, significantly more than endorsements of harm-based explanations (20%; $\chi^{2}\left(1\right)=257,P<0.0001$), fairness-based explanations (32%; $\chi^{2}\left(1\right)=164,P<0.0001$) and utility maximization-based explanations (9%; $\chi^{2}\left(1\right)=369,P<0.0001$). Conversely, universalization was endorsed less strongly for the nonthreshold cases (52% for harm, $\chi^{2}\left(1\right)=56,P<0.0001$; 29% for fairness, $\chi^{2}\left(1\right)=180,P<0.0001$; 33% for utility maximization; $\chi^{2}\left(1\right)=153,P<0.0001$). This suggests that universalization is invoked both consistently and selectively for threshold cases. Subjects did, however, consider each of the other moral explanations to be valid for the specific category we had predicted a priori (90% for harm; 87% for fairness; 74% for utility maximization; binomial tests, all P<0.0001). We then replicated this study, achieving a similar pattern of results (*SI Appendix*).

In sum, participants recognized universalization as a good explanation for why an action is wrong in threshold problems. They consider it a better explanation in these cases than appeals to harm, fairness or utility maximization. For other categories of moral violation, however, they reliably select these other categories of moral judgment.

## Study 2: Testing the Characteristic Features of Universalization Judgments

Next, we ask whether participants’ moral judgments exhibit the key qualitative patterns predicted by the model of universalization described above. We use threshold problems as a testing ground because the predictions of universalization are different from those made by the alternate models described in the Introduction. Universalization’s most distinctive prediction is that moral permissibility judgments in threshold problems will depend on whether 1) the number of interested parties $n_{i}$ (i.e., those who would act under the absence of moral constraint) exceeds 2) the harm threshold (i.e., where the utility function $U\left(n_{i}\right)$ takes on a negative slope). If it does, universalizing moral liberty would decrease utility, and so the logic of universalization dictates that the action in question is morally wrong. In order to probe these factors, we designed a case in which the numbers of interested parties and the utility function are precisely specified for participants.

### Study 2a: Sensitivity to “Interested Parties.”

We begin by testing a distinctive and central feature of universalization: It is sensitive to the number of “interested parties.” These are people who would hypothetically choose to perform an action if they felt morally at liberty to do so.

Participants read about a threshold problem arising in a small lakeside vacation town. Twenty vacationers currently fish in a sustainable way, but then a new fishing hook becomes available that allows each vacationer to catch many more fish. If fewer than three vacationers start using the hook, there will be no negative consequences; if more than seven vacationers start using the hook, then there is guaranteed to be a total collapse of the fish population by summer’s end. Thus, the harm threshold occurs at three to seven interested parties. The protagonist of this vignette, John, is interested in using the new hook. Participants are asked if doing so would be morally acceptable.

Our critical manipulation is the number of interested parties, that is, people who are actually interested in catching more fish. John knows this number because he speaks to each one of the other vacationers individually. In the low-interest condition, none of the other vacationers are interested in the new hook (i.e., $n_{i}=0$, below the harm threshold): They feel like they have enough fish and enjoy fishing at a leisurely pace. Thus, moral permission to use the hook can be universalized without harm. In the high-interest condition, all of the other vacationers would be interested in using the new hook and catching more fish ($n_{i}=19$, above the harm threshold), and yet each of them has personally decided against it because they are worried about sustainability. Thus, moral permission to use the hook would be harmful if universalized. For this reason, universalization predicts that participants will judge John’s action less acceptable in the high-interest condition than in the low-interest condition.

Crucially, however, in both versions of the vignette, John knows that none of the other vacationers will use the new hook. He can therefore safely start using the hook without any actual negative consequences, because one person using the hook is below the harm threshold. An outcome-based model therefore predicts that John’s action should be acceptable. John also knows that there is no “rule” in his community against using the hook. We confirmed that participants agreed with these premises about rules and outcomes (see *SI Appendix* for analysis of these and other control questions).

**Fig. 4.**
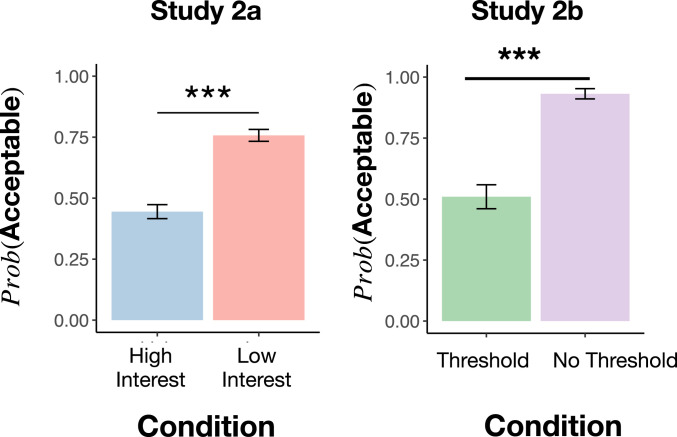
Study 2 results. Study 2a: There is a significant difference between the high-interest and low-interest conditions, as predicted if participants use universalization to make moral judgments in this case (but not predicted by an outcome- or rule-based model). Study 2b: There is a significant difference between the threshold and no-threshold conditions, as predicted by universalization (but not by a norms-based model). Error bars are SEM. *** indicates P<0.001.

Finally, to ensure that our finding was robust, we constructed and tested four additional contexts with similar structure. Rather than fishing in a lake, the contexts involved stories where a group of people were foraging for mushrooms, hunting birds, trapping rabbits, or gathering clams. We found similar results across all scenarios (*SI Appendix*). Here, analysis is collapsed across contexts (Fig. 4).

As predicted, subjects judged John’s action to be significantly more morally acceptable in the low-interest condition than in the high-interest condition (low interest: 76%; high interest: 44%; $\chi^{2}\left(1\right)=62.0,P<0.001$, two-tailed, $V_{Cramer}=0.32,{CI}_{95\%}\left[0.25,0.40\right],n=608$; Fig. 4).

Further, we showed that participants explicitly endorse universalization as a good explanation for why John’s behavior is wrong in the high-interest case (just as they do for other moral threshold problems tested in study 1). And, just as in study 1, participants endorse universalization (86%) significantly more than harm (25%), utility maximization (24%), or fairness (56%) (*SI Appendix*).

In summary, we find that more people consider John’s action morally acceptable when other relevant parties are disinterested. This pattern of judgment is predicted by the logic of universalization, but not by standard theories of outcome- or rule-based moral judgment.

Whether it can be explained by a norm-based model is more ambiguous. We attempted to write our scenarios so that there was no overt expression of moral norms correlated with the number of interested parties (31). Nevertheless, in the context of our experiments, “interested parties” who say that they would like to perform an action but have chosen not to (perhaps because they consider it morally wrong) may establish a relevant descriptive norm (in other words, participants may approximate $n_{p}$ as $n_{i}$ in our cases; see Eq. **5**). Thus, a norm-based mechanism of moral judgment could explain the sensitivity to the number of interested parties without any role for universalization. We designed study 2b to provide a strong test of the norm versus universalization models.

### Study 2b: Sensitivity to the Presence of a Harm Threshold.

We next focused on a prediction of universalization that is not shared with norm-based influences on moral judgment. Like norms, our universalization model is sensitive to people’s behavior. But, unlike norms, it is also sensitive to the consequences of their collective behavior—to the shape of the utility function U(n). For this reason, threshold problems provide a natural testing ground to distinguish between the norm-based model and universalization. In a threshold problem, universalization predicts that, if the number of interested parties exceeds the harm threshold, we should observe a drop in moral permissibility. But, if there is no harm threshold (and, more generally, no decrease in utility as more people act), then universalization predicts the action will be judged morally permissible. The norms model, in contrast, is not sensitive to the presence or absence of a harm threshold. Rather, it is only sensitive to the proportion of people who act or do not act. Thus, in study 2b, we manipulate the presence vs. absence of a harm threshold while holding the number of interested nonactors constant. Universalization predicts an effect of this manipulation, while the norms model does not.

In the “threshold” condition, we described the same fisheries case as in study 2a, specifying that the fish population would collapse if more than five people adopted the new hook, and that all 20 of the vacationers say they would be interested in using the new hook in the absence of moral constraint. However, they are each personally committed to traditional fishing methods, and therefore will not use the new hook. Moreover, the fishermen are not even aware of the harm threshold; therefore they cannot be acting on a norm generated by the presence of the threshold. (We also replicated this finding in two modified cases: one in which the fishermen are aware of the harm threshold and one in which the fishermen simply say that they think using the new hook is wrong, without further justification; *SI Appendix*.) In the “no threshold” condition, we describe the same setup except that we state that there is no threshold beyond which the fish population would be harmed. In other words, this condition has an entirely different utility structure: one without a harm threshold or any utility decrease as the number of people acting increases. Here, our model of universalization predicts that participants should judge it permissible for John to use the hook.

Crucially, however, a norm-based model should not distinguish between these two conditions. In both conditions, John surveys his neighbors, and, in both conditions, an identical number of neighbors say that they will not use the new hook because of their personal moral commitment to traditional fishing practices.

As predicted by the universalization model, more participants judged the “no threshold” case permissible (93%) than judged the “threshold case” permissible (51% [$\chi^{2}\left(1\right)=58.61,P<0.001$, two-tailed, $V_{Cramer}=0.48,{CI}_{95\%}\left[0.36,0.60\right],n=250$]; Fig. 4 and *SI Appendix*).

## Discussion

Across these two studies, we find that participants’ judgments match the key qualitative prediction of universalization. People were more likely to judge an action wrong if, when universalizing the action, the number of interested parties who would hypothetically act exceeds a threshold sufficient to trigger worse outcomes. We showed this by manipulating the number of interested parties while holding the harm threshold constant (study 2a) and then by manipulating the harm threshold while holding the number of interested parties constant (study 2b). This distinctive pattern of sensitivity is not easily explained by standard models of moral judgment that appeal to rules, outcomes, or norms.

While our data are consistent with some participants employing the logic of universalization, they also clearly show that not all participants do so. For instance, even in the “high-interest” case, 44% of participants judged that it was permissible for John to act in study 2a. The judgments of these participants are most consistent with an outcome- or rule-based mechanism of moral judgment. As a rough estimate of the frequency of universalization in our sample, we can compute the difference in proportions between conditions: about 32% of participants for study 2a, and about 35% in study 2b. We probe these individual differences more carefully in study 4 below.

## Study 3: Whose Utilities Matter?

Universalization asks what the outcome would be if everyone felt at liberty to act in a certain way. But whose utilities count?

One possibility is that people are concerned with the welfare of everyone involved—in our fisheries case, for instance, all of the fishermen. This wide scope of concern is a hallmark of utilitarian moral theories (including rule consequentialism, e.g., ref. 17), which tend to be concerned with impartial maximization of aggregate utility. A wide scope is also predicted by contractualist theories that project the outcome of ideal bargaining and negotiation. On such theories, the welfare of everyone involved will influence the bargain (not through simple maximization of aggregate utility, but through taking everyone’s perspective into account, e.g., refs. 21–23).

Alternatively, moral judgment may reflect concerns about whether universalizing an action has a bad impact on the actor’s own personal utility. This may occur if participants approach the question from John’s perspective, assuming that he is motivated (or adapted, by biological or cultural evolution) to identify a moral rule that maximizes his personal interests when universally applied.

There is also precedent in philosophy for the view that universalization hinges on considering the (hypothetical) impact on the actor of universalizing his action. A range of scholars working in the Kantian tradition suggest that we should condemn actions that undermine their own goal once universalized (21, 22, 32–34). For instance, John wants to use the new, powerful fishing hook as a means of catching extra fish, but, if the fish population disappears (as would happen if everyone used the new hook), using the new hook will no longer enable John to achieve his goal.

Thus, in study 3, we designed a case that sharply dissociates the impact on John from that on the other fishermen. We modified the case used in study 2 so that John operates a motorized tour boat and can increase his profits by using a new kind of motor oil. Using this motor oil can also increase the profits of the fishermen. John’s utility $U_{j}$ is unaffected by how many other people (i.e., fishermen) use the motor oil. Thus, $U_{j}\left(20\right)>U_{j}\left(0\right)$. By contrast, the fisherman’s utility $U_{f}$ decreases if too many people use the new motor oil because it will destroy the fish population. Thus, $U_{f}\left(20\right)<U_{f}\left(0\right)$.

If participants’ moral judgments depend on what happens to everyone’s utility when the action is universalized, they should judge this new “tour boat” case identically to the original case used in study 2. Although John would not personally be harmed if everybody started using the new motor oil, the other fishermen would. On the other hand, if participants are narrowly concerned with the impact on John when the action is universalized, they should judge this new tour boat case quite differently. Since John suffers no harm from adoption of the new motor oil, and his goal is not undermined, universalizing the action should be deemed permissible (whether or not there is high or low interest among fishermen).

**Fig. 5.**
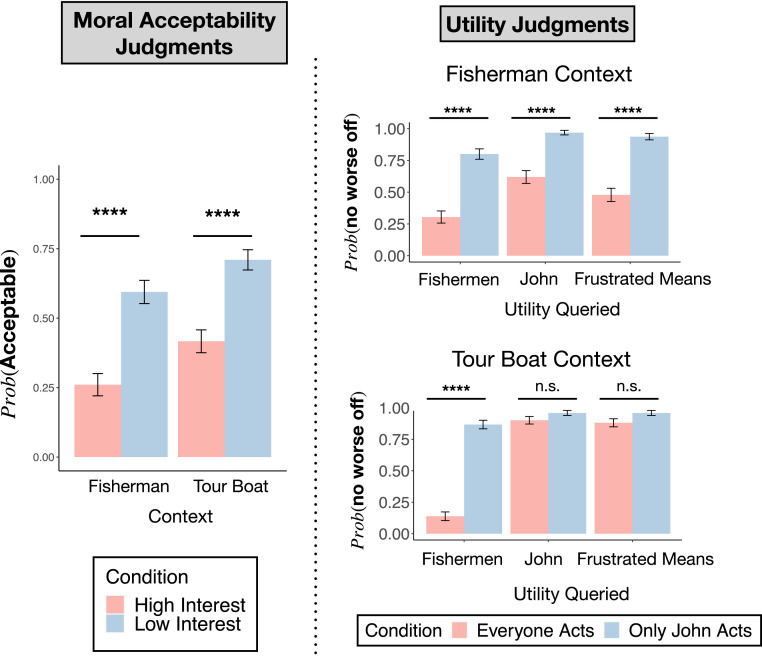
(*Left*) Moral acceptability judgments for the fisherman and tour boat contexts. Each context exhibits a similar pattern of moral acceptability: The action is more permissible in the low-interest condition compared to the high-interest condition. (*Right*) Utility measures for each context. In the fisherman context, all three measures (everyone’s utility, John’s utility, and frustrated means) show a similar pattern. This context therefore does not differentiate between the measures. In the tour boat context, the measures exhibit very different patterns. In this context, it becomes clear that considering everyone’s utility explains the moral acceptability data better than considering John’s utility in isolation or whether John’s action is a means to his end. Error bars are SEM. **** indicates P<0.0001; n.s. indicates P>0.05.

We asked participants to make moral judgments of the high- and low-interest conditions of the original “fisherman” case and the modified “tour boat” case in a 2×2 between-subjects design. As Fig. 5, *Left* shows, we observed a large effect of condition (high interest vs. low interest) in both cases. We analyzed these data with a logistic regression, which shows a significant effect of condition on subjects’ judgments (P<0.0001), a significant main effect of context (P<0.001), but no condition-by-case interaction (P=0.59). These results suggest that people did not attend selectively to John’s utility but, instead, broadly to the utility of both John and the fishermen.

In order to confirm that participants perceived the utility functions for John and for the fishermen as we intended, a separate group read the same scenarios but provided ratings on the utility consequences for various parties. Specifically, for each case, they rated the difference in utility for John if everyone decided to use the new motor oil compared to no one using it, $U_{j}\left(20\right)-U_{j}\left(0\right)$ (subjects responded “better off,” “worse off,” or “the same”); the difference in utility for the rest of the fishermen, $U_{f}\left(20\right)-U_{f}\left(0\right)$ (again, as “better off,” “worse off,” or “the same”); and the likelihood that John would be able to bring about his goal (“more likely than before,” “less likely than before,” or “the same as before”, testing Kant’s concept of a frustrated means). While subjects chose between three options, in Fig. 5, *Right* we report the more illustrative metric of the percentage of subjects that reported that the relevant parties would be no worse off (i.e., U(20)−U(0)>0; we determine this by collapsing the answers of “better off” and “the same”). See *SI Appendix* for full data.

As expected, in the context where John is a fisherman, there are significant and large differences across the conditions both when subjects are asked about the utility of the fishermen (everyone acts: M = 0.30, SE = 0.048; John acts: M = 0.80, SE = 0.041; $\chi^{2}\left(1\right)$ Yates’ corrected = 44.5, ϕ=0.49, P<0.0001), John’s utility (everyone acts: M = 0.48, SE = 0.052; John acts: M = 0.94, SE = 0.025; $\chi^{2}\left(1\right)$ Yates’ corrected = 45.7, ϕ=0.49, P<0.0001), and whether John’s action would no longer be a means to his goal (everyone acts: M = 0.62, SE = 0.051; John acts: M = 0.97, SE = 0.018; $\chi^{2}\left(1\right)$ Yates’ corrected = 33.0, ϕ=0.42, P<0.0001). Specifically, when everyone uses the new hook, most subjects judged that John and the fishermen would be worse off. In contrast, when only John uses the new hook, most participants judged that John and the fishermen would be no worse off. In contrast, in the tour boat context, when we ask subjects about John’s utility, the difference between the conditions is only marginally significant, with a small effect size (everyone acts: M = 0.88, SE = 0.032; only John acts: M = 0.96, SE = 0.020; $\chi^{2}\left(1\right)$ Yates’ corrected = 3.11, P=0.078; ϕ=0.12). We see a similar pattern of findings when we ask whether John’s action would no longer be a means to his goal (everyone acts: M = 0.90, SE = 0.030; only John acts: M = 0.96, SE = 0.020; $\chi^{2}\left(1\right)$ Yates’ corrected = 1.76, P=0.18; ϕ=0.094). In contrast, when subjects are asked about everyone’s utility, the difference between conditions is large and significant (everyone acts: M = 0.14, SE = 0.034; only John acts: M = 0.87, SE = 0.034; $\chi^{2}\left(1\right)$ Yates’ corrected = 102.8, P<0.0001, ϕ=0.72) (see Fig. 5). Notably, the 8% of subjects who felt that John would be worse off if everyone adopted the new oil is not sufficient to explain the 29% of subjects that treat the cases differently when asked about moral acceptability judgments, because the upper bound on the difference in the conditions for John’s utility (95% CI upper bound = 0.13) is smaller than the lower bound on the difference across the conditions for moral judgment (95% CI lower bound = 0.216; high interest: M = 0.417, SE = 0.041, low interest: M = 0.710, SE = 0.036). This same logic extends to the 6% difference in the conditions when subjects are asked about John’s action being a means to his goal.

In summary, when applying universalization, people consider a wide scope of utilities. In our cases, they are concerned not just with the utility of John, or with Kant’s conception of self-undermining action, but, instead, they are concerned with the utility of all those who use the lake. In *Discussion*, we return to consider what this implies about the nature and function of universalization.

## Study 4: Testing a Computational Model of Universalization Judgments

So far, we have tested the key qualitative predictions generated by our model of universalization. Next, we rely on this model to generate and test its quantitative predictions. In study 4a, we test the model’s predictions about how fine-grained shifts in the model’s key parameters, $n_{i}$ and U(n), affect moral judgment. We also use these data to more precisely characterize individual differences in moral judgment and determine the prevalence of universalization in our test population. Next, in study 4b, we test whether incorporating a measure of participants’ subjective perception of the utility function will improve the model’s quantitative fit to the data.

### Study 4a: Fine-Grained Predictions of the Universalization Model.

We begin by collecting a richer dataset on participant moral judgments in the fisherman case. We employ a design that uses more fine-grained manipulation of both the utility function, U, (building on study 2b) and the number of interested parties (building on study 2a).

**Fig. 6.**
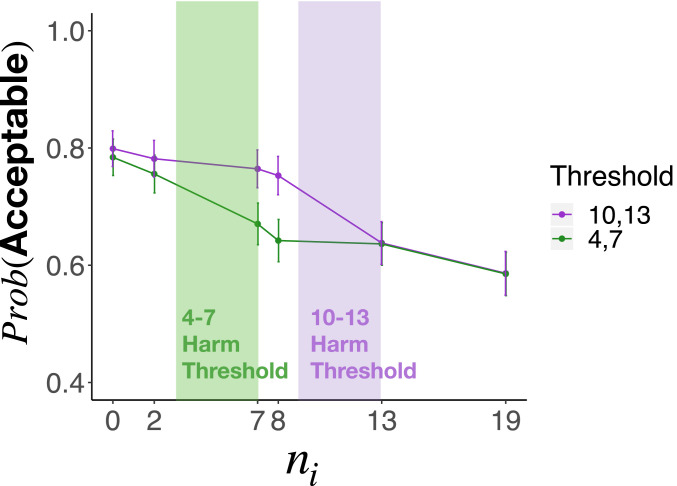
Moral acceptability judgments for study 4. The probability of a subject judging the action morally acceptable as a function of the number of people interested in using the new hook. The location of the threshold (indicated by shaded areas) impacts moral permissibility judgments, suggesting that the way that the utility aggregates impacts moral permissibility. This distinguishes universalization from a norm-based account of moral judgment.

Specifically, we include seven levels of interested parties $n_{i}\in \left[0,2,7,8,13,19\right]$. We also varied harm threshold: In the “low-threshold” condition, this threshold occurred between 4 (harm) and 7 (collapse), while, in the “high-threshold” condition, it occurred between 10 (harm) and 14 (collapse). The results track the key signatures of our model. Moral permissibility drops the fastest at the point where the number of interested parties exceeds the harm threshold (Fig. 6). In other words, permissibility is not a simple function of interested parties or the harm threshold alone; it depends upon their interaction. We use this distinctive prediction of universalization to compare it quantitatively to alternative models, and to characterize individual differences in patterns of moral judgment.

#### Comparison to the norms model.

Although both the universalization and norms models can explain why moral judgment is sensitive to the number of interested parties $n_{i}$ in our cases (assuming participants approximate $n_{p}$ as $n_{i}$), only universalization predicts that the effect of $n_{i}$ additionally depends on the structure of the utility function $U\left(n_{i}\right)$. We exploited this feature in study 2b. Here, using the data from study 4a, we again contrast the universalization and norms models. Specifically, we compared a linear mixed effects model (which includes fixed effects for $n_{i}$, the location of the harm threshold, and their interaction) with a model excluding both the location of the harm threshold and its interaction with $n_{i}$. (We included participants as a random effect and specified maximal random slopes.) The full model is significantly preferred ($\chi^{2}=8.64,P=0.013$), suggesting that universalization is a better predictor of these data than the norms model.

#### Comparison to the rules model.

As a further test of the possibility that people infer moral rules from the behavior of interested nonactors, we asked subjects whether any rule prohibited using the new hooks. We analyzed their rule judgments with a logistic regression with $n_{i}$ as the predictor. Although there was a marginal effect of the number of interested parties on rule perception (z=1.798;P=0.072), when rule judgments and $n_{i}$ were entered into a logistic regression to predict moral judgments, rule judgments were not a significant predictor of moral judgments (z=0.037;P=0.97), nor was their interaction with the number of interested parties (z=−0.038;P=0.97).

#### Individual differences in patterns of moral judgment.

**Fig. 7.**
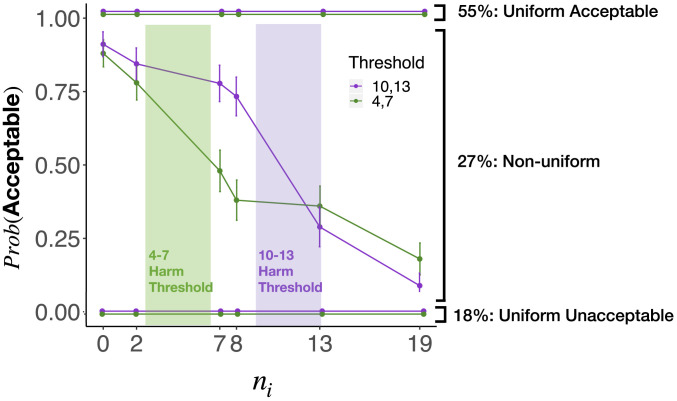
Moral acceptability judgments for study 4, broken down into three response patterns: subjects who uniformly considered the action acceptable, those who uniformly considered the action unacceptable, and those with nonuniform response patterns. This latter group exhibits the pattern predicted by universalization.

We next used this repeated measures design to estimate the proportion of participants employing different methods of moral judgment. Outcome- and rule-based models of moral judgments make the distinctive prediction that participants will render a uniform pattern of judgment across all six values of $n_{i}$ that we tested in study 2 (Fig. 2*B*). Consistent with these predictions, we found that 55% of participants judged John’s action uniformly morally acceptable, while 18% judged it uniformly unacceptable. Fig. 7 plots the moral acceptability judgments of the remaining 27% of participants whose judgments were nonuniform. These participants clearly exhibit the pattern associated with universalization, with the predominant decrease in moral acceptability occurring at the harm threshold specific to each condition. We provide a more precise quantitative fit of our model to these participants’ judgments in study 4b. We elaborate on the implications of these findings for our theory in *Discussion*.

### Study 4b: Improving Quantitative Model Fit—Measuring Empirical Utility Functions.

So far, we have assumed that participants represent the utility function U precisely as it is described in our scenarios (Fig. 1*A*). On this assumption, utility is flat until the harm threshold, drops quickly in the threshold range, and then is low and flat beyond it.* Presumably, however, participants’ subjective impressions of the utility function deviate somewhat from this idealization. For example, if subjects know that utility will precipitously fall at n = 4, they may be uncertain about whether it actually remains constant between n = 3 and 4, and thus impute a negative slope to the utility function before the threshold. In this study, we empirically estimate the utility function imputed by participants. Our experiment focuses on the regions before and after the threshold range, about which our stimuli were most explicit. We then ask whether substituting this empirical estimate for the idealized function improves the fit of our universalizing model (as it should if participants are universalizing with respect to their subjective utility functions) (35).

We asked a new group of participants to read our stimuli and rate the utility outcomes at various settings of n. Specifically, participants read the stories from study 4a, either for high-threshold or low-threshold conditions in a between-subjects design. We asked them how much better or worse things would go for all of the fishermen if various numbers of people (n∈[1,3,8,9,14,20]) started to use the new hook, as compared to the status quo in which nobody is using it (i.e., U(n)−U(0)). They responded on a scale from −50 (“a lot worse off”) to 50 (“a lot better off”), where 0 indicates no change.^†^

**Fig. 8.**
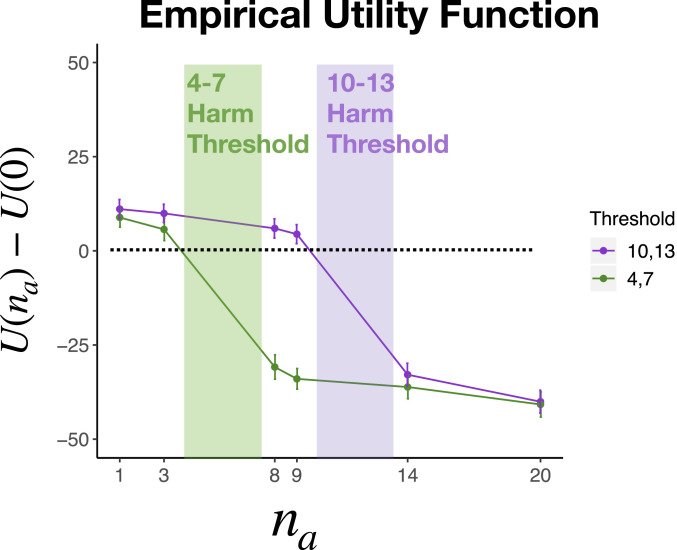
Subjective utility functions produced by the subjects in study 4 for both the 4,7 and 10,13 threshold conditions. These utility functions deviate from our idealized function in that utility slopes downward rather than being completely flat before and after the threshold regions.

As Fig. 8 shows, participants generally felt that things would go slightly better if a below-threshold number of people began to use the hook. This makes sense: Things should be better for that minority of hook users, and no worse for the nonusers. And, generally, participants felt that things would go much worse if an above-threshold number of people began to use the hook. This also makes sense: Above the threshold, the fish population collapses, and things go worse for everyone. Notably, however, the empirical utility function does not conform precisely to any idealized utility function, insofar as it shows slightly negative slopes both above and below the critical threshold (perhaps reflecting subjective uncertainty about the location of the threshold, as described above).

### Applying the Empirical Utility Function.

**Fig. 9.**
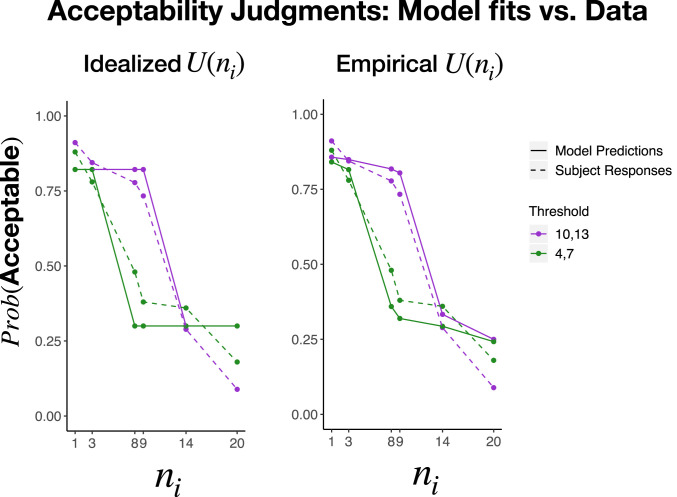
Moral judgments predicted by the universalization model (solid lines) overlaid against the observed moral acceptability data (dotted lines) for those participants who exhibit not-uniform patterns of judgment. (*Left*) The model based on the ideal utility curve. (*Right*) The model based on the empirical utility curve.

We next assessed whether the universalization model achieves a superior fit to moral acceptability data when we substitute the empirical utility function obtained above in place of the idealized utility function assumed so far. If our theory is correct, it should. In order to do this, we fit participants’ moral judgment data from study 4 to our formal model of universalization (i.e., Eq. **1**) by optimizing the values of the parameters τ and β. We only included data from the 27% of participants who gave a nonuniform pattern of judgments across values of the number of interested parties $n_{i}$ (Fig. 9), since these are the only subjects who show evidence of applying universalization to these cases. We generated an empirically estimated value of $U\left(n_{i}\right)$ by entering the mean of the data described in the preceding section (i.e., the values plotted in Fig. 8). We generated idealized values of $U\left(n_{i}\right)$: we set U(n)=0 for all $n_{i}$ less than the threshold and U(n)=1 for all $n_{i}$ at or above the threshold. The critical feature of this idealized model is simply that the values before and after the threshold should be constant (not, for instance, that they be symmetric around a utility midpoint; see *SI Appendix* for further details).

Fig. 9 shows the predicted moral acceptability judgments of each model overlaid on the data obtained from study 4. Overall, we find a strong correspondence between the model predictions and the data. It is especially striking that we find a strong correspondence between the model and the data across our two different harm threshold conditions, given that we fit identical parameter values to both. In other words, the difference between these conditions is not explicit in the model, but rather derives exclusively from the shape of the utility functions: participants’ perceptions of the $U\left(n_{i}\right)$ for different harm thresholds. We also find that the model fit when using empirical utility functions (Akaike information criterion, AIC=609) is substantially better than the model fit when using an idealized utility function (AIC=621). This finding supports the inference that subjects use universalization as a method of moral decision-making by showing that subjects’ subjective perceptions of the relevant utility functions best predict moral judgments.

Having obtained an empirical utility function that differs slightly from our idealized assumed utility function, it is important to ask whether it can improve the predictive power of any of our alternative models of moral judgment. It obviously will not improve the predictive power of rule- or norm-based models, since these do not involve the utility function at all. But what about outcome-based models, which do?

Applying the outcome-based model described in Eq. **2** to the threshold problems we are investigating here, the key question is how the utility of one person acting compares with the utility of nobody acting, that is, whether U(1)≥U(0). The slope of our idealized utility function is flat in this range, and so, according to Eq. **2**, it is permissible for John to begin using the hook. But, our empirically derived utility function shows that some participants (14.6%) actually believe that one person using the hook will result in a negative utility. According to our outcome-based model, such participants would therefore be predicted to judge it impermissible for John to start using the hook. This is consistent with the observation in study 2 that 18% of participants judged John’s action impermissible uniformly, for any number of interested parties—a pattern of judgment inconsistent with universalization, but consistent with an outcome-based model applied to the empirical utility function. (Of course, this outcome-based model is insensitive to the number of interested parties, and thus cannot explain the substantial proportion of participants whose moral acceptability judgments depend on it.)

## Study 5: Universalization across Development

Kohlberg (27) famously described moral development as a progression through six stages of reasoning. The sixth and highest stage of moral development is the ability to provide moral justifications that appeal to the abstract concept of universalization. Kohlberg (28) argued that “Without formal moral theory men naturally attain to a ‘stage 5’ [the penultimate moral stage].” While we agree that universalization receives its fullest treatment in formal philosophical theories, in this study, we investigate whether the seeds of this sophisticated reasoning process are present in some form from a young age.

Kohlberg diagnosed his subjects’ moral stage by classifying the justifications they provided for their moral judgments—their ability to explicitly introspect on their moral cognition—rather than by attending to patterns of judgments themselves. Since Kohlberg, a rich body of work has shown that young children and even preverbal infants exhibit a sophisticated moral sense that is not necessarily dependent on their ability to linguistically communicate their reasoning (36–39). Yet, while we have learned much about the development of moral cognition by studying the judgment patterns of young children, we know far less about whether children use universalization to make moral judgments.

We presented children aged 4 y to 11 y, and a small group of adults, with two stories that were similar in structure to the fisheries cases used in studies 1 through 4. In one story, for instance, there is a path made of rocks that the kids in the story like to walk on to get home. Jimmy has a rock collection and would like to take one of the rocks for his collection. In the low-interest condition, Jimmy is the only one who wants to take a rock, and therefore moral license to take a rock can be universalized without harm. In the high-interest condition, all of the kids have rock collections and would like to take a rock. However, they have all decided not to take the rocks because they want to path to stay intact. In this case, universalizing permission to take the rocks would cause the path to disappear.

In these stories, the actor is described as completely alone when he acts, so that children will not assume that his behavior will influence others. We also make it clear that no other kids are going to do the action, confirming comprehension with a series of control questions (*SI Appendix*).

## Results

**Fig. 10.**
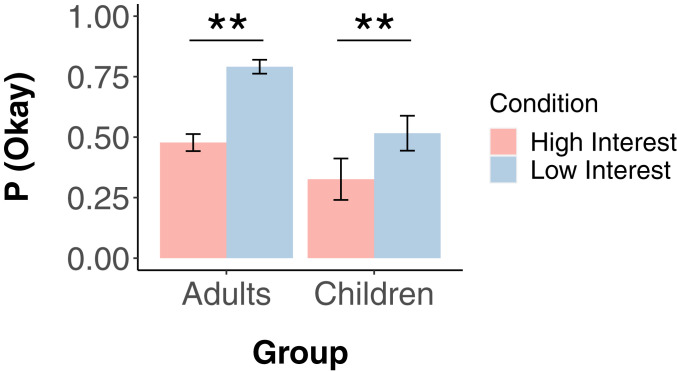
Probability children and adults judging the story ”Okay” in study 5. There is a significant difference between the high-interest and low-interest cases for both adults and children. Error bars are SEM. ** indicates *P* < 0.01.

The results from the adult sample replicate the findings in study 2a: The high-interest condition is judged to be significantly more permissible than the low-interest condition ($\left(\chi^{2}\left(1\right)=42.3,P\;=<\;0.0001\right)$ (Fig. 10).

Each child received two stories in counterbalanced order, one low interest and one high interest. Five children opted to hear only one story. For those who answered both, 143 (79.4%) subjects gave the same answer to both stories: 74 subjects (41.1%) said not Okay to both stories, and 69 subjects (38.3%) said Okay to both stories. Of those who gave different answers to the two stories, 26 (14.4%) gave the expected pattern of answers (permissible in low interest, impermissible in high interest), and only 11 (6.1%) gave the opposite pattern (McNemar’s test of changes P=0.02). Because so few subjects switched their answers, however, the remainder of the analysis was conducted on answers to the first story only.

The central finding (Fig. 10) is that children were significantly more likely to judge the low-interest case permissible compared to the high-interest case ($\chi^{2}\left(1\right)=6.85,P=0.009$, two-tailed, $V_{Cramer}=0.19,{CI}_{95\%}\left[0.06,0.34\right],n=185$, Bayes factor = 5.58 in favor of $H_{1}$). Moreover, this pattern holds for the entire age range we tested (4 y to 11 y old). To assess potential developmental trends, we compared three models of the data: one including condition only, one including the main effect of age, and one including an age × condition interaction. In the latter two models, there is no significant effect of age or the age × condition interaction, and the data are best explained by the model that includes only condition (*SI Appendix*).

## Discussion

Across five studies, we show that both adults and children sometimes make moral judgments well described by the logic of universalization, and not by standard outcome-, rule-, or norm-based models of moral judgment. We model participants’ judgment of the moral acceptability of an action as proportional to the change in expected utility in the hypothetical world where all interested parties feel free to do the action. This model accounts for the ways in which moral judgment is sensitive to the number of parties hypothetically interested in an action, the threshold at which harmful outcomes occur, and their interaction. By incorporating data on participants’ subjectively perceived utility functions, we can predict their moral judgments of threshold problems with quantitative precision, further validating our proposed computational model. These data suggest a new and intriguing correspondence between ordinary people’s moral judgments and common features of diverse philosophical theories which all draw on the logic of universalization (15–17, 21, 25).

Our findings contribute to current theories of moral judgment in several ways. First, many of these theories emphasize the role of relatively simple, intuitive heuristics (5, 10, 40, 41). Our results suggest a highly structured and complex cognitive architecture that contributes to moral judgment. Second, a dominant view in moral development holds that young children are incapable of universalization-based judgment (28). In contrast, we find its fingerprints in young children’s patterns of judgments. (These results must be interpreted with caution, however: The experiment we conducted with children does not test the full range of our model’s predictions, nor does it include the full range of manipulation checks that we employed with adults.) Third, many current theories of moral psychology focus on the twin contributions of outcome- and rule-based moral judgment (5, 12, 13). We offer universalization as a key element of the moral mind that composes elements of outcome- and rule-based theories in a distinctive way.

Our work raises important questions about the cognitive mechanisms supporting universalization. The philosophical literature includes several related, yet distinct, versions of universalization, each corresponding to a viable cognitive model. For instance, people might ask, “Which rule would be best for us all?” (more akin to rule utilitiarian theories; e.g., ref. 17), or, instead, “Which rule would everyone agree to?” (more akin to contractualist theories; e.g., ref. 23), and so on. Distinguishing these possibilities is an important direction for future research.

Another important question is how people infer the relevant general principle to universalize from the observation of a single person’s single act. For instance, if, on July 17, John uses the new fishing hook, is the rule to be universalized “Everybody may use the hook,” or “Everybody named John may use the hook,” or “Everybody may use the hook on July 17,” etc.? This echoes the general problem of inductive inference: In this case, given the output of a policy or rule, how can we infer its underlying generative structure (42, 43)?

Other important questions concern the scope of universalization: How widely is it employed? We used threshold problems in our studies because universalization uniquely predicts the moral judgments for these cases; no other prominent theory of moral psychology (including utility-based, rule-based, and norm-based theories, which we formally contrast to our model) correctly predicts how we respond to threshold cases. However, the question remains: Is universalization used in other kinds of social dilemmas (such as aggregation problems)? Which cases elicit universalization-based reasoning?

Moreover, although many participants in our studies employed universalization when faced with threshold problems, many used different strategies for moral judgment. These other participants may instead have relied on norm-, outcome-, or rule-based strategies, or others yet to be described. If the choice of strategy is a stable individual difference, what demographic or cultural factors can predict it? Alternately, it is possible that a single subject may sometimes use universalization and sometimes a different strategy to make moral judgments for threshold problems. If this is the case, subjects are then faced with a “strategy selection problem” (44). How do they know which strategy for moral judgment to use when? Can we manipulate the cases to encourage participants to adopt one strategy over another? As study 1 shows, there are some cases where universalization is the wrong strategy to apply. It is not wrong to punch a person because “What if everybody did that?”; likewise with betraying a friend, french kissing a sibling, or compensating an employee unfairly. Following most contemporary theories of moral psychology, we assume that human moral judgments are generated by multiple complementary mechanisms. We have demonstrated a key place for universalization among this set.

Finally, what is the relationship between universalization and these other mechanisms of moral judgment? They might be entirely independent. In contrast to this possibility, however, we suggest that universalization is intimately related to both outcome- and rule-based mechanisms. Specifically, universalizing identifies rules that would be good candidates for everyone to agree on: When impartially applied, they tend to improve utility. It is, in short, metamoral rule: It endorses rules that bring about good outcomes.

In this respect, universalization is a cognitive mechanism that achieves outcomes similar to social processes that generate moral norms—ones such as negotiation, bargaining, and cultural or biological evolution (4). For instance, when negotiating, people will typically agree upon arrangements that are both fair and ensure mutual benefit (19, 23, 24, 45, 46). As we show in study 3, universalization is sensitive to the welfare outcomes for all interested parties, and not just the welfare of the actor. This may reflect a simple aggregation of social welfare (as in utilitarian theories), but it might, instead, reflect a more complex bargaining solution (as in contractualist theories). In other words, when universalizing, people may simulate a virtual bargaining process (20) to determine which moral liberties and constraints everybody would agree to. Our study cannot speak directly to this possibility, but it stands out as an important direction for future research. Similarly, the logic of universalization mirrors some key concepts in evolutionary game theory. In general, biologically or culturally evolved moral norms should be stable equilibria, in the sense that nobody can improve their position by unilateral defection (47). But, when there are several such viable equilibria, which should we expect to observe? All else being equal, evolutionary dynamics favor those yielding greater aggregate payoffs (48). Thus, biologically or culturally evolved moral norms tend toward payoff-maximizing rules that everyone would agree to (49). In sum, universalization can generate moral norms similar to social processes such as negotiation and bargaining, or cultural and biological evolution. Unlike these processes, however, universalization can be quickly and efficiently implemented within a single person’s mind.

As these connections illustrate, when we judge an action by universalizing, we receive not just a judgment of the action but also a new rule. This suggests that, just as people can collectively establish new rules (4), they can also individually employ universalization when no clear moral rule exists. For instance, having decided that John shouldn’t use his more powerful fishing hooks in this instance, we have established a new candidate rule: “Don’t use the hooks.” Once derived, a rule affords both cognitive efficiency (50) and consistency. Rules established by applying the logic of universalization may be reused across an individual’s life and transmitted across generations as a cultural inheritance. In this manner, the logic of universalization holds the power to transform individuals’ outcome-based preferences into impartial and binding moral rules for the community.

The Harvard University IRB approved these studies. Informed consent was given by all adult subjects and by legal guardians of children.

### Study 1.

One hundred fifty subjects participated in this study, recruited from Amazon MTURK through turkprime and paid a small amount for their participation. Twenty subjects were excluded for failing an attention check. Subjects read 12 short stories, each involving a moral violation. Subjects were asked to indicate whether they thought that each of four explanations was a convincing explanation for why that action was wrong.

### Study 2a.

Preregistration site is http://aspredicted.org/blind.php?x=fx3kz7. One thousand subjects participated in this study, recruited from MTURK through turkprime and paid a small amount for their participation; 394 subjects were excluded for failing control questions. A different group of 200 subjects were recruited to make explanation judgments. The procedure for this part of the study was similar to that of study 1, except that subjects read the high-interest fishing scenario used in study 2a; 60 subjects were excluded for failing an attention check. Subjects were randomly assigned to one of two conditions, which varied the reason that the fishermen chose to abstain from using the powerful fishing hook. For details, see *SI Appendix*.

### Study 2b.

Preregistration site is https://aspredicted.org/blind.php?x=hd589d. Four hundred thirty-one subjects participated in this study, recruited from MTURK through turkprime and paid a small amount for their participation; 181 subjects were excluded for failing control questions (*SI Appendix*).

### Study 3.

One thousand two hundred forty-two subjects participated in this study, recruited from MTURK through turkprime and paid a small amount for their participation. Subjects were divided into two groups: the moral judgment group (*n* = 840) and the expected utility group (*n* = 402; two additional subjects were accidently allowed to take the experiment after our 400 subject cap); 284 subjects were excluded from the study for failing one or more control questions in the moral judgment group, and 16 subjects were excluded in the expected utility group. Subjects in both groups were randomly assigned to one context (fisherman or tour boat) and one condition (high interest or low interest). Subjects in the moral judgment group answered different questions about the scenarios than did subjects in the expected utility group. See *SI Appendix* for further details.

### Study 4a.

Preregistration site is https://aspredicted.org/blind.php?x=c44jr2. Seven hundred subjects participated in this study, recruited from MTURK through turkprime and paid a small amount for their participation; 350 subjects were excluded for failing control questions. Subjects were randomly assigned to one of two conditions. The 4,7 condition is as follows: Up to four people can use the new hook with no effect on the fish population; once seven people use the new hook, the fish population will go extinct. The 10,13 condition is as follows: Up to 10 people can use the new hook with no effect on the fish population; once 13 people use the new hook, the fish population will go extinct. Each subject was told that *N* people are interested in using the new hook. Subjects answered a series of questions about the story. Subjects then read the same story, the only change being that a new value of *N* was given. *N* was chosen at random without replacement from the following values until all values of *N* were been seen by each subject: 0, 2, 7, 8, 13, 19. See *SI Appendix* for further details.

### Study 4b.

Preregistration site is http://aspredicted.org/blind.php?x=at7cs8. Three hundred subjects participated in this study, recruited from MTURK through turkprime and paid a small amount for their participation; 18 subjects were excluded from the study for failing control questions. Subjects were randomly assigned to one of two conditions (4,7 condition and 10,13 condition, as described in study 4a) and one of three questions (yielding a 2 × 3 design). The three questions were everyone’s expected utility, John’s expected utility, and frustrated means (*SI Appendix*). Only the data from the everyone’s expected utility condition are reported in the main text. Results from the remaining two conditions are reported in *SI Appendix*. Each subject read the story and was asked what would happen if *N* subjects used the new hook (exact wording varied depending on the utility curve; *SI Appendix*). *N* was chosen at random without replacement from the following values until all values of *N* were seen by each subject: 0, 2, 7, 8, 13, 19. (This list is for John’s utility and frustrated means. For everyone’s utility, subjects see N+1, i.e., 1, 3, 8, 9, 14, 20.)

### Study 5.

Children (ages 4 y to 11 y) were recruited in the Boston Common. We planned to analyze our data using a Bayesian analysis to avoid having to plan for a specific stopping rule, due to our uncertainty about the effect size for this study and the difficulty of recruiting subjects (51–54). In *Results*, we report the Bayes factor as the main item of analysis, although we also include *P* values to conform with current standards for data reporting. Ultimately, 191 subjects were included in the analysis (mean age = 7.5 y); 28 additional children were recruited but excluded from the analysis for failing the screening or control questions. Children were first told simple stories accompanied by pictures to verify their competence with English and to ensure that they could use ”Okay” and ”not Okay” to make simple moral judgments. Subjects who did not answer these questions correctly were excluded from the analysis. Following the screening, children heard two test stories accompanied by pictures, counterbalancing condition and context. See *SI Appendix* for complete stimuli. Two hundred one adult subjects received the same stimuli as children. Adults were recruited from MTURK through turkprime and were paid a small amount for their participation (*SI Appendix*).

## Supplementary Material

Supplementary File

## Data Availability

Human subjects data have been deposited in Github (https://github.com/sydneylevine/universalization).

## Footnotes

*Or perhaps slightly increasing, given that the people who act are slightly better off while nobody else is harmed.

^†^This experiment also contained two additional conditions (John’s expected utility and frustrated means), which queried the change in utility in different ways. These ways of measuring utility produce results very similar to the ones we report here, so the results from the remaining two conditions are reported in *SI Appendix*.
