# Supplementary material for: The logic of universalization guides moral judgment
Source: Proc Natl Acad Sci U S A. 2020 Oct 2;117(42):26158–69. doi: 10.1073/pnas.2014505117 (PMC7584905; doi:10.1073/pnas.2014505117)
Supplement: Supplementary File [file pnas.2014505117.sapp.pdf]

PNAS
www.pnas.org

Supplementary Information for

Universalization reasoning guides moral judgment

Sydney Levine, Max Kleiman-Weiner, Laura Schulz, Joshua Tenenbaum, Fiery Cushman

Sydney Levine
E-mail: smlevine@mit.edu

This PDF file includes:

Figs. S1 to S12
Tables S1 to S2
SI References

www.pnas.org/cgi/doi/10.1073/pnas.2014505117

Data Availability

Data and analysis scripts for all studies are available at github.com/sydneylevine/universalization.

Study 1

Materials. 150 subjects participated in this study, recruited from Amazon MTURK through turkprime and were paid a small amount for their participation. 20 subjects were excluded for failing an attention check. Subjects read 12 short stories, each involving a moral violation. Subjects were asked to indicate if they thought that each of four explanations was a convincing explanation for why that action was wrong.

Subjects were presented with the following twelve stories in a randomized order:

1. Threshold Problems:
    (a) Ali and his family are on a hike. They stop to eat lunch next to a stream. There is a group of apple trees near the stream. Sometimes the apples from the apple trees roll into the stream and that's OK. But if too many apples end up in the stream, then the ducks will eat them all and get sick. Lots of people hike on this trail every day. Ali picks an apple and eats it for lunch. He doesn't want to carry the apple core with him. So he throws it into the stream.
    (b) Betty is visiting a museum. She is looking at a very old book. If lots of people touch the pages of the book, then over a long time the dirt from their hands will make the book so dirty that no one will be able to read it any more. Betty decides to turn the pages of the book.
    (c) It is story time in Camen's class. The teacher starts reading a story out loud and all the kids in the class are quietly listening to the story. Camen has something he wants to ask. Camen asks his question without raising his hand.
2. Utility Maximization Problems:
    (a) Liana has an extra snack today that she doesn't want. Lenny doesn't have a snack at all and asks if he can have Liana's extra one. Liana throws her extra snack in the trash.
    (b) Martin realizes that the roof in his classroom is leaking and the water is about to drip on everyone's art projects. Jenny's art project is very large, so it is being kept in the closet and is not going to get wet. But Martin realizes that if he gets Jenny's project out of the closet, he can use it to protect everyone else's project, even though Jenny's will be destroyed. Martin decides to let the water drip on everyone's projects and they are all ruined except Jenny's.
    (c) Nick sees that the window to the classroom is open and that everyone's homework is about to blow away. Nick picks up his own homework and moves it to safe place, but leaves everyone else's homework there. Everyone else's homework blows away.
3. Fairness Problems:
    (a) It is Oscar's turn to give out stickers to the three kids in his group. There are three stickers Oscar can give out. Oscar gives one sticker to Sam, two stickers to John, and no stickers to Emily.
    (b) Penelope's class makes a mess while they are painting. The teacher lets everyone go outside for recess except for Penelope who has to clean up the whole mess.
    (c) Quinn brings cookies to share with his class. He decides to only give the cookies to the boys.
4. Harm Problems:
    (a) Phil got angry at his friend and punched him in the face.
    (b) Jane performed a song in the talent show. Anne told her that her performance was terrible.
    (c) Mark is allergic to peanuts. David knows this, but he gives him a salad that has peanuts in it anyway.

After each scenario, subjects were asked to indicate if each of four explanations was a convincing explanation for why that action was wrong. (See Fig. S1 below.)

The four explanations were as follows:

1. Because that person could have helped more people.
2. Because that was unfair.
3. Because if everyone did that, the outcome would be bad.
4. Because that harmed someone.

Attention check: At the end of the survey, subjects read the following: "Thanks for taking the time to do this survey. This final question is just here to confirm you are paying attention. Please do not answer this question (do not check any of the boxes). Instead, write 'I am paying attention' in the box labeled 'Other' below. Thanks so much for your help!" Subjects were presented with the following options: High School, Associate's Degree, Bachlor's Degree, Professional Degree or PhD, Some College. Subjects were excluded if they checked any of the levels of education or failed to write "I am paying attention" in the free-response box.

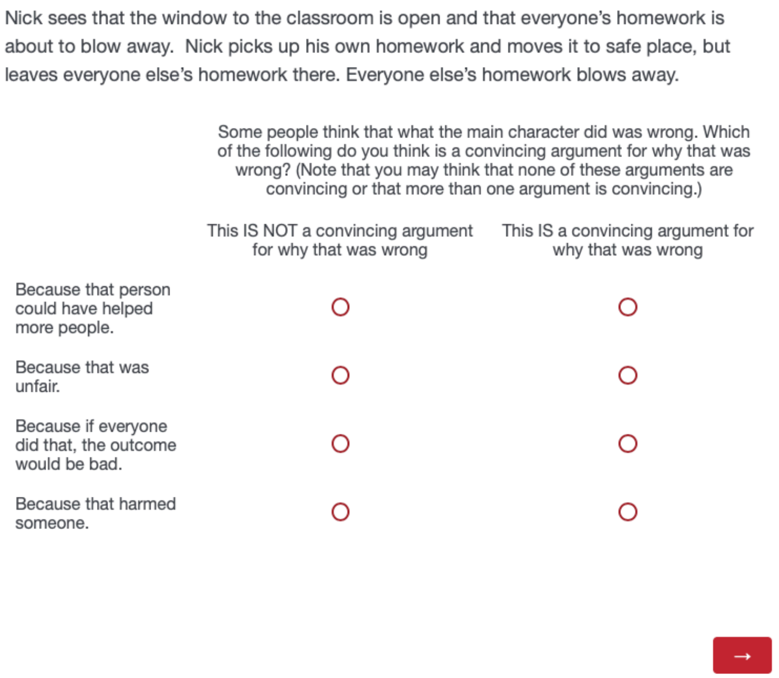

Nick sees that the window to the classroom is open and that everyone's homework is about to blow away. Nick picks up his own homework and moves it to safe place, but leaves everyone else's homework there. Everyone else's homework blows away.

Some people think that what the main character did was wrong. Which of the following do you think is a convincing argument for why that was wrong? (Note that you may think that none of these arguments are convincing or that more than one argument is convincing.)

	This IS NOT a convincing argument for why that was wrong	This IS a convincing argument for why that was wrong
Because that person could have helped more people.	○	○
Because that was unfair.	○	○
Because if everyone did that, the outcome would be bad.	○	○
Because that harmed someone.	○	○

→

Fig. S1. Options seen by subjects in Study 1.

Study 1 Replication

Study 1 was replicated using identical stimuli. A similar pattern of results was achieved, as reported below. The only change to the stimuli was as follows. In the Study reported in the main manuscript, subjects were told after each vignette, "Some people think that what the main character did was wrong. Which of the following do you think is a convincing argument for why that was wrong? (Note that you may think that none of these arguments are convincing or that more than one argument is convincing.)" In the replication, the parenthetical instruction was absent.

Materials. 150 subjects participated in this study, recruited from Amazon MTURK through turkprime and were paid a small amount for their participation. 21 subjects were excluded for failing an attention check.

A. Results. As predicted, and in line with what we found in Study 1 in the main manuscript, participants strongly preferred universalization to explain why an individual action is wrong in a threshold problem (Figure S2). 74% of responses to the threshold problems indicated that universalization was a good explanation of moral wrongness in that case, significantly more than endorsements of harm-based explanations (24%; $\chi^2(1) = 190, p < .0001$), fairness-based explanations (34%; $\chi^2(1) = 326, p < .0001$) and utility-maximization-based explanations (16%; $\chi^2(1) = 260, p < .0001$). Conversely, universalization was endorsed less-strongly for the non-threshold cases (45% for harm, $\chi^2(1) = 65, p < .0001$; 33% for fairness, $\chi^2(1) = 126, p < .0001$; 36% for utility-maximization; $\chi^2(1) = 110, p < .0001$). This suggests that universalization is invoked both consistently and selectively for threshold cases. Subjects did, however, consider each of the other moral explanations to be valid for the specific category we had predicted *a priori* (82% for harm; 83% for fairness; 69% for utility maximization; Binomial tests, all $p's < .0001$).

1. Study 2a

A. Moral Judgments.

B. Materials. This study was preregistered (see http://aspredicted.org/blind.php?x=fx3kz7). 1000 subjects participated in this study, recruited from Amazon MTURK through turkprime and were paid a small amount for their participation. 394 subjects were excluded for failing control questions. Subjects were randomly assigned to to the High Interest or the Low Interest Condition and to one of the five contexts (birds, clams, fish, rabbits, mushrooms). Each subject read and responded to one story only.

Exclusion criteria (each question is modified slightly to fit the appropriate context): 1. How many people, besides John, would like to use the new hooks if there were no bad effects of doing so?

To be included in the study, participants must report 19 in the high interest condition and 0 in the low interest condition.

2. How many people regularly fish in Lake Wilson in the summer?

To be included, subjects must answer 19, 20, or 21.

3. How many people, besides John, are actually going to use the new hooks?

To be included, subjects must answer 0.

4. Will it make a difference to the fish population if John uses the new hooks?

There are three possible answers to this question: (A) It will make the fish population more healthy (B) It will make the fish population less healthy (C) It will not make a difference to the fish population. To be included, subjects must answer (C).

Subjects read the following instructions prior to beginning the study: "On the following pages you will be asked to read a short story and answer questions about it. The questions on each page of the survey will be different, but the story will remain the same. (The story will appear on each page for your reference.) After the survey there will be an opportunity to let us know if something was confusing or unclear."

Subjects read the following stimuli. On each new page, the story appeared again for subjects' reference. Below, we include the text of the stimuli used across the five contexts in the High Interest and Low Interest conditions. For the first story only, we present all the questions asked to subjects. After that, we present only the text of the scenarios. The questions were identical from context to contesxt with the exception of small changes relevant to the context change (eg, "fish" is replaced by "clams). For the first two stories, we bold the differences between the High Interest and the Low Interest conditions for the ease of the reader. Subjects did not see the text in bold.

Context: Clams

Condition: High Interest

–Page 1–

Wilson Bay is a small bay on the coast of Oregon. Each summer, a few dozen families move into small cottages near the bay for the season. The vacationers enjoy boating, swimming, and digging for razor clams in the bay and they've gotten to know each other over the course of many summers together.

Most people who like to dig for clams in Wilson Bay catch about a dozen clams each day and cook them for dinner. These people buy their clamming gear from a small shop on the main road. Up until now, the shop has sold one kind of digging device, a clam tube made out of plastic. But it has recently starting selling a new kind of clam tube made out of aluminum. The new tube allows people to find many more clams than they would with the older tube. There are 20 people who regularly dig for clams in Wilson Bay during the summer. If 7 or more people started using the new tube for clamming, then by the end of the summer, the entire clam population would collapse and there would be no more clams in Wilson Bay ever again. None

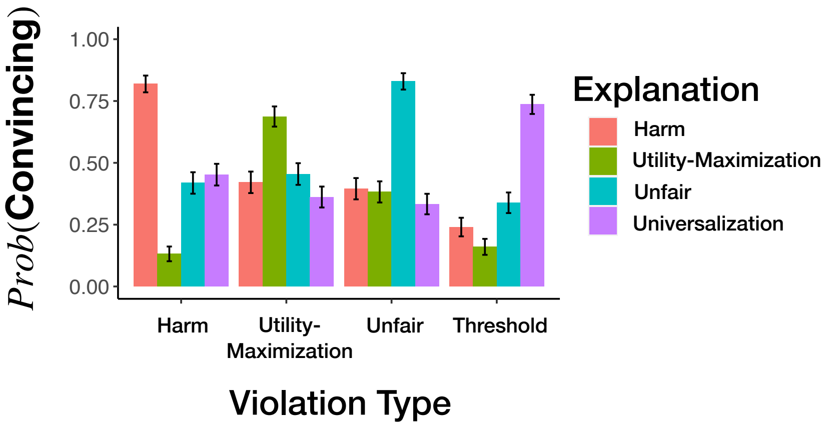

Fig. S2. Results from the Study 1 replication. The data show the same pattern as those reported in Study 1 in the main manuscript. Subjects endorse universalization as an explanation for threshold problems preferentially over harm-based, fairness-based, and utility-based explanations. Likewise, those explanations are selected for the appropriate moral violations. Error bars are standard error of the mean.

of the vacationers would want that to happen. However, up to 3 people can use the new tube with no decrease at all in the size and health of the clam population. Those people will be able to catch more clams more quickly, everyone else will still be able to catch the same amount of clams as before, and the clam population would continue to be exactly as large and as healthy as in past years.

It is impossible to tell what tube someone is using from a distance, so if someone starts using the new tube, no one else will know about it. No one has bought the new tube yet, however. John would like to use the new tube because he would like to catch more clams faster. He is thinking about whether or not to buy the new tube. John wants to find out what his neighbors think about the new tube, so he decides he is going to visit the cottages of all the people who go clamming in Wilson Bay and ask them. John manages to talk to all 20 people who regularly go clamming in the Bay. **When John sits down with each person to talk about the new tube, each person says something like this: "I would love to use that new tube to catch more clams faster, but what would happen if everyone did that? If everyone used the new tube, all the clams would disappear from the bay. I don't want that to happen, so I'm not going to use the new tube." John thinks to himself: No one else is going to use the new tube. Under these circumstances, it's OK for me to use the new tube and catch more clams.** [Note: Bold added here to distinguish between High Interest and Low Interest Conditions. Participants did not see the text in bold.]

Please answer the following comprehension questions, so we know that you read the story.

If John starts using the new tube, how many people will know about it? [Free response]

How many people, besides John, would like to use the new tube if there were no bad effects of doing so? [Free response.]

How many people regularly dig for clams in Wilson Bay in the summer? [Free response.]

How many people, besides John, are actually going to use the new tube? [Free response.]

–Page 2–

Is it morally acceptable for John to use the new tube? [Yes/No]

–Page 3–

Is there a rule on the bay that the new tube is forbidden? [Yes/No]

–Page 4–

Will it make a difference to the clam population if John uses the new tube? [It will make the clam population more healthy / It will make the clam population less healthy / It will not make a difference to the clam population]

Context: Clams

Condition: Low Interest

Wilson Bay is a small bay on the coast of Oregon. Each summer, a few dozen families move into small cottages near the bay for the season. The vacationers enjoy boating, swimming, and digging for razor clams in the bay and they've gotten to know each other over the course of many summers together.

Most people who like to dig for clams in Wilson Bay catch about a dozen clams each day and cook them for dinner. These people buy their clamming gear from a small shop on the main road. Up until now, the shop has sold one kind of digging device, a clam tube made out of plastic. But it has recently starting selling a new kind of clam tube made out of aluminum. The new tube allows people to find many more clams than they would with the older tube. There are 20 people who regularly dig for clams in Wilson Bay during the summer. If 7 or more people started using the new tube for clamming, then by the end of the summer, the entire clam population would collapse and there would be no more clams in Wilson Bay ever again. None of the vacationers would want that to happen. However, up to 3 people can use the new tube with no decrease at all in the size and health of the clam population. Those people will be able to catch more clams more quickly, everyone else will still be able to catch the same amount of clams as before, and the clam population would continue to be exactly as large and as healthy as in past years.

It is impossible to tell what tube someone is using from a distance, so if someone starts using the new tube, no one else will know about it. No one has bought the new tube yet, however. John would like to use the new tube because he would like to catch more clams faster. He is thinking about whether or not to buy the new tube. John wants to find out what his neighbors think about the new tube, so he decides he is going to visit the cottages of all the people who go clamming in Wilson Bay and ask them. John manages to talk to all 20 people who regularly go clamming in the Bay. **When John sits down with each person to talk about the new tube, each person says something like this: "I'm just not interested in using that new tube. I really only need to catch a few clams a day, and I like to do that at a leisurely pace and spend all day clamming. I'm not in any rush and I don't need to catch more clams. Besides, if everyone used the new tube, all the clams would disappear from Wilson Bay. I don't want that to happen. But even if there were so many clams in the bay that everyone could use the new tube, I wouldn't want to use it anyway."** [Note: Bold added here to distinguish between High Interest and Low Interest Conditions. Participants did not see the text in bold.] John thinks to himself: No one else is going to use the new tube. Under these circumstances, it's OK for me to use the new tube and catch more clams.

Fish Context

High Interest Condition

Lake Wilson is a small lake in upstate New York. Each summer, a few dozen families move into small cottages near the lake for the season. The vacationers enjoy boating, swimming, and fishing in the lake and they've gotten to know each other over the course of many summers together. Most people who like to fish in Lake Wilson catch a few fish each day and cook them for dinner. These people buy their fishing gear from a small tackle shop on the main road. Up until now, the tackle shop has sold

one kind of fishing hook. But it has recently starting selling a new kind of hook that allows people to catch many more fish than they would with the older hook. There are 20 people who regularly fish in Lake Wilson during the summer. If 7 or more people started using the new hook for fishing, then by the end of the summer, the entire fish population would collapse and there would be no more fish in Lake Wilson ever again. None of the vacationers would want that to happen. However, up to 3 people can use the new hooks with no decrease at all in the size and health of the fish population. Those people will be able to catch more fish more quickly, everyone else will still be able to catch the same amount of fish as before, and the fish population would continue to be exactly as large and as healthy as in past years. It is impossible to tell what hook someone is using from a distance, so if someone starts using the new hooks, no one else will know about it. No one has bought the new hooks yet, however. John would like to use the new hooks because he would like to catch more fish faster. He is thinking about whether or not to buy the new hooks. John wants to find out what his neighbors think about the new hooks, so he decides he is going to visit the cottages of all the people who fish in Lake Wilson and ask them. John manages to talk to all 20 people who regularly fish in the lake. When John sits down with each person to talk about the new hooks, each person says something like this: "I would love to use those new hooks to catch more fish faster, but what would happen if everyone did that? If everyone used the new hooks, all the fish would disappear from the lake. I don't want that to happen, so I'm not going to use the new hook." John thinks to himself: No one else is going to use the new hooks. Under these circumstances, it's OK for me to use the new hooks and catch more fish.

Fish Context

Low Interest Condition

Lake Wilson is a small lake in upstate New York. Each summer, a few dozen families move into small cottages near the lake for the season. The vacationers enjoy boating, swimming, and fishing in the lake and they've gotten to know each other over the course of many summers together. Most people who like to fish in Lake Wilson catch a few fish each day and cook them for dinner. These people buy their fishing gear from a small tackle shop on the main road. Up until now, the tackle shop has sold one kind of fishing hook. But it has recently starting selling a new kind of hook that allows people to catch many more fish than they would with the older hook. There are 20 people who regularly fish in Lake Wilson during the summer. If 7 or more people started using the new hook for fishing, then by the end of the summer, the entire fish population would collapse and there would be no more fish in Lake Wilson ever again. None of the vacationers would want that to happen. However, up to 3 people can use the new hooks with no decrease at all in the size and health of the fish population. Those people will be able to catch more fish more quickly, everyone else will still be able to catch the same amount of fish as before, and the fish population would continue to be exactly as large and as healthy as in past years. It is impossible to tell what hook someone is using from a distance, so if someone starts using the new hooks, no one else will know about it. No one has bought the new hooks yet, however. John would like to use the new hooks because he would like to catch more fish faster. He is thinking about whether or not to buy the new hooks. John wants to find out what his neighbors think about the new hooks, so he decides he is going to visit the cottages of all the people who fish in Lake Wilson and ask them. John manages to talk to all 20 people who regularly fish in the lake. When John sits down with each person to talk about the new hooks, each person says something like this: "I'm just not interested in using those new hooks. I really only need to catch a few fish a day, and I like to do that a leisurely pace and spend all day fishing. I'm not in any rush and I don't need to catch more fish. Besides, if everyone used the new hooks, all the fish would disappear from the lake. I don't want that to happen. But even if there were so many fish in the lake that everyone could use the new hooks, I wouldn't want to use them anyway." John thinks to himself: No one else is going to use the new hooks. Under these circumstances, it's OK for me to use the new hooks and catch more fish.

Rabbits Context

High Interest Condition

Stonyville is a small forested town in upstate New York. Each summer, a few dozen families move into small cottages in Stonyville for the season. The vacationers enjoy boating, swimming, and trapping rabbits in the forest of Stonyville and they've gotten to know each other over the course of many summers together. Most people who like to trap rabbits in the Stonyville forest catch about a few rabbits each day and cook them for dinner. These people buy their traps from a small hunting shop on the main road. Up until now, the shop has sold one kind of trap. But it has recently starting selling a new kind of trap that allows people to catch many more rabbits than they would with the older trap. There are 20 people who regularly trap rabbits in the Stonyville forest during the summer. If 7 or more people started using the new traps for hunting, then by the end of the summer, the entire rabbit population would collapse and there would be no more rabbits in the Stonyville forest ever again. None of the vacationers would want that to happen. However, up to 3 people can use the new traps with no decrease at all in the size and health of the rabbit population. Those people will be able to catch more rabbits more quickly, everyone else will still be able to catch the same amount of rabbits as before, and the rabbit population would continue to be exactly as large and as healthy as in past years. It is impossible to tell what trap someone is using from a distance, so if someone starts using the new traps, no one else will know about it. No one has bought the new traps yet, however. John would like to use the new traps because he would like to catch more rabbits faster. He is thinking about whether or not to buy the new traps. John wants to find out what his neighbors think about the new trap, so he decides he is going to visit the cottages of all the people who trap rabbits and ask them. John manages to talk to all 20 people who regularly trap rabbits in the Stonyville forest. When John sits down with each person to talk about the new traps, each person says something like this: "I would love to use those new traps to catch more rabbits faster, but what would happen if everyone did that? If everyone used the new traps, all the rabbits would disappear from the forest. I don't want that to happen, so I'm not going to use the new traps." John thinks to himself: No one else is going to use the new traps. Under these circumstances, it's OK for me to use the new traps and catch more rabbits.

Rabbits Context

Low Interest Condition

Stonyville is a small forested town in upstate New York. Each summer, a few dozen families move into small cottages in Stonyville for the season. The vacationers enjoy boating, swimming, and trapping rabbits in the forest of Stonyville and they've gotten to know each other over the course of many summers together. Most people who like to trap rabbits in the Stonyville forest catch about a few rabbits each day and cook them for dinner. These people buy their traps from a small hunting shop on the main road. Up until now, the shop has sold one kind of trap. But it has recently starting selling a new kind of trap that allows people to catch many more rabbits than they would with the older trap. There are 20 people who regularly trap rabbits in the Stonyville forest during the summer. If 7 or more people started using the new traps for hunting, then by the end of the summer, the entire rabbit population would collapse and there would be no more rabbits in the Stonyville forest ever again. None of the vacationers would want that to happen. However, up to 3 people can use the new traps with no decrease at all in the size and health of the rabbit population. Those people will be able to catch more rabbits more quickly, everyone else will still be able to catch the same amount of rabbits as before, and the rabbit population would continue to be exactly as large and as healthy as in past years. It is impossible to tell what trap someone is using from a distance, so if someone starts using the new traps, no one else will know about it. No one has bought the new traps yet, however. John would like to use the new traps because he would like to catch more rabbits faster. He is thinking about whether or not to buy the new traps. John wants to find out what his neighbors think about the new trap, so he decides he is going to visit the cottages of all the people who trap rabbits and ask them. John manages to talk to all 20 people who regularly trap rabbits in the Stonyville forest. When John sits down with each person to talk about the new traps, each person says something like this: "I'm just not interested in using that new traps. I really only need to catch a few rabbits a day, and I like to do that at a leisurely pace and spend all day hunting. I'm not in any rush and I don't need to catch more rabbits. Besides, if everyone used the new traps, all the rabbits would disappear from the forest. I don't want that to happen. But even if there were so many rabbits in the forest that everyone could use the new traps, I wouldn't want to use it anyway." John thinks to himself: No one else is going to use the new traps. Under these circumstances, it's OK for me to use the new traps and catch more rabbits.

Birds Context

High Interest Condition

Stonyville is a small forested town in upstate New York. Each summer, a few dozen families move into small cottages in Stonyville for the season. The vacationers enjoy hiking, camping, and hunting pheasants in the forest of Stonyville and they've gotten to know each other over the course of many summers together. Most people who like to hunt shoot a few birds each day and cook them for dinner. These people buy their hunting gear from a small hunting shop on the main road. Up until now, the hunting shop has sold one kind of gun. But it has recently starting selling a new kind of gun that allows people to shoot more accurately and therefore hunt many more pheasants than they would with the older gun. There are 20 people who regularly hunt for pheasants in the Stonyville forest during the summer. If 7 or more people started using the new gun for hunting, then by the end of the summer, the entire pheasant population would collapse and there would be no more pheasants in Stonyville ever again. None of the vacationers would want that to happen. However, up to 3 people can use the new gun with no decrease at all in the pheasant population. Those people will be able to hunt more pheasants more quickly, everyone else will still be able to hunt the same amount of pheasants as before, and the pheasant population would continue to be as healthy as in past years. It is impossible to tell what gun someone is using from a distance, so if someone starts using the new gun, no one else will know about it. No one has bought the new gun yet, however. John would like to use the new gun because he would like to hunt more pheasants faster. He is thinking about whether or not to buy the new gun. John wants to find out what his neighbors think about the new gun, so he decides he is going to visit the cottages of all the people who hunt pheasants and ask them. John manages to talk to all 20 people who regularly hunt in Stonyville. When John sits down with each person to talk about the new gun, each person says something like this: "I would love to use that new gun to hunt more pheasants faster, but what would happen if everyone did that? If everyone used the new gun, all the pheasants would disappear from the forest. I don't want that to happen, so I'm not going to use the new gun." John thinks to himself: No one else is going to use the new gun. Under these circumstances, it's OK for me to use the new gun and hunt more pheasants.

Birds Context

Low Interest Condition

Stonyville is a small forested town in upstate New York. Each summer, a few dozen families move into small cottages in Stonyville for the season. The vacationers enjoy hiking, camping, and hunting pheasants in the forest of Stonyville and they've gotten to know each other over the course of many summers together. Most people who like to hunt shoot a few birds each day and cook them for dinner. These people buy their hunting gear from a small hunting shop on the main road. Up until now, the hunting shop has sold one kind of gun. But it has recently starting selling a new kind of gun that allows people to shoot more accurately and therefore hunt many more pheasants than they would with the older gun. There are 20 people who regularly hunt for pheasants in the Stonyville forest during the summer. If 7 or more people started using the new gun for hunting, then by the end of the summer, the entire pheasant population would collapse and there would be no more pheasants in Stonyville ever again. None of the vacationers would want that to happen. However, up to 3 people can use the new gun with no decrease at all in the pheasant population. Those people will be able to hunt more pheasants more quickly, everyone else will still be able to hunt the same amount of pheasants as before, and the pheasant population would continue to be as healthy as in past years. It is impossible to tell what gun someone is using from a distance, so if someone starts using the new gun, no one else will know about it. No one has bought the new gun yet, however. John would like to use the new gun because he would like

to hunt more pheasants faster. He is thinking about whether or not to buy the new gun. John wants to find out what his neighbors think about the new gun, so he decides he is going to visit the cottages of all the people who hunt pheasants and ask them. John manages to talk to all 20 people who regularly hunt pheasants in the Stonyville forest. When John sits down with each person to talk about the new gun, each person says something like this: “I’m just not interested in using that new gun. I really only need to hunt a few pheasants a day, and I like to do that at a leisurely pace and spend all day hunting. I’m not in any rush and I don’t need more pheasants Besides, if everyone used the new gun, all the pheasants would disappear from the forest. I don’t want that to happen. But even if there were so many pheasants in the forest that everyone could use the new gun, I wouldn’t want to use it anyway.” John thinks to himself: No one else is going to use the new gun. Under these circumstances, it’s OK for me to use the new gun and hunt more pheasants.

Mushrooms Context

High Interest Condition

Stonyville is a small forested town in upstate New York. Each summer, a few dozen families move into small cottages in Stonyville for the season. The vacationers enjoy hiking, camping, and foraging for mushrooms in the forest of Stonyville and they’ve gotten to know each other over the course of many summers together. Most people who like to forage for mushrooms gather a basket of mushrooms each day and cook them for dinner. These people buy their foraging gear from a small shop on the main road. Up until now, the shop has sold one kind of mushroom foraging knife. But it has recently starting selling a new kind of knife that allows people to gather mushrooms more quickly and therefore get many more mushrooms than they would with the older knife. There are 20 people who regularly forage for mushrooms in Stonyville during the summer. If 7 or more people started using the new knife for foraging, then by the end of the summer, the entire mushroom population would collapse and there would be no more mushrooms in Stonyville ever again. None of the vacationers would want that to happen. However, up to 3 people can use the new knife with no decrease at all in the mushroom population. Those people will be able to gather more mushrooms more quickly, everyone else will still be able to gather the same number of mushrooms as before, and the mushroom population would continue to be as healthy as in past years. It is impossible to tell what knife someone is using from a distance, so if someone starts using the new knife, no one else will know about it. No one has bought the new knife yet, however. John would like to use the new knife because he would like to gather more mushrooms faster. He is thinking about whether or not to buy the new knife. John wants to find out what his neighbors think about the new knife, so he decides he is going to visit the cottages of all the people who forage for mushrooms and ask them. John manages to talk to all 20 people who regularly forage in the Stonyville. When John sits down with each person to talk about the new knife, each person says something like this: “I would love to use that new knife to gather more mushrooms faster, but what would happen if everyone did that? If everyone used the new knife, all the mushrooms would disappear from the forest. I don’t want that to happen, so I’m not going to use the new knife.” John thinks to himself: No one else is going to use the new knife. Under these circumstances, it’s OK for me to use the new knife and gather more mushrooms.

Mushrooms Context

Low Interest Condition

Stonyville is a small forested town in upstate New York. Each summer, a few dozen families move into small cottages in Stonyville for the season. The vacationers enjoy hiking, camping, and foraging for mushrooms in the forest of Stonyville and they’ve gotten to know each other over the course of many summers together. Most people who like to forage for mushrooms gather a basket of mushrooms each day and cook them for dinner. These people buy their foraging gear from a small shop on the main road. Up until now, the shop has sold one kind of mushroom foraging knife. But it has recently starting selling a new kind of knife that allows people to gather mushrooms more quickly and therefore get many more mushrooms than they would with the older knife. There are 20 people who regularly forage for mushrooms in Stonyville during the summer. If 7 or more people started using the new knife for foraging, then by the end of the summer, the entire mushroom population would collapse and there would be no more mushrooms in Stonyville ever again. None of the vacationers would want that to happen. However, up to 3 people can use the new knife with no decrease at all in the mushroom population. Those people will be able to gather more mushrooms more quickly, everyone else will still be able to gather the same number of mushrooms as before, and the mushroom population would continue to be as healthy as in past years. It is impossible to tell what knife someone is using from a distance, so if someone starts using the new knife, no one else will know about it. No one has bought the new knife yet, however. John would like to use the new knife because he would like to gather more mushrooms faster. He is thinking about whether or not to buy the new knife. John wants to find out what his neighbors think about the new knife, so he decides he is going to visit the cottages of all the people who forage for mushrooms and ask them. John manages to talk to all 20 people who regularly forage in the Stonyville. When John sits down with each person to talk about the new knife, each person says something like this: “I’m just not interested in using that new knife. I really only need to gather one basket of mushrooms a day, and I like to do that at a leisurely pace and spend all day foraging. I’m not in any rush and I don’t need to gather more mushrooms. Besides, if everyone used the new knife, all the mushrooms would disappear from the forest. I don’t want that to happen. But even if there were so many mushrooms in the forest that everyone could use the new knife, I wouldn’t want to use it anyway.” John thinks to himself: No one else is going to use the new knife. Under these circumstances, it’s OK for me to use the new knife and gather more mushrooms.

C. Moral Judgments: Supplemental Results. In the main text, we report the data collapsed across scenario context. Here we break the data down by context and look for an effect of context on moral judgment. We compared three logistic regressions to predict answers to moral permissibility judgments. Model 1 includes only condition as a predictor. Model 2 includes context as well as condition. Model 3 includes the context x condition interaction. Model 1 fits the data best on AIC and BIC measures.

Model 2 shows that there is no main effect of context. Model 3 shows that there is no significant interaction with context. See Fig. S4 for model specifications and comparison statistics. Model comparisons were conducted in R with the package ggstatsplot (1). Data is graphed by context in Fig. S3.

As indicated in the preregistration document, we anticipated that answers to the "rule" and "knowledge" control questions would not explain the effect of condition on subjects' moral judgments. There were only two subjects (across all contexts) who answered that there was a rule on the lake forbidding the use of the hook. Judgments about the presence of a rule, therefore, do not explain subjects' moral permissibility judgments.

For the knowledge question, we expected the majority of subjects to answer that no one would know about John using the new hook (as indicated in the vignette). However, some subjects reasonably assumed that the person who sold John the hook would know about it or that knowledge of John using the hook would inevitably spread (they sometimes indicated this in the free-response comment box at the end of the study). Do assumptions about knowledge of John's use of the new hook differ across the conditions in a way that could possibly explain our findings? This seems unlikely for several reasons. First, we analyzed the knowledge question by looking at what proportion of subjects in each condition judged that no one would find out about John using the new hook (as opposed to thinking that 1 or more people would find out about it). There is a small but significant difference across the conditions, but the difference goes in the opposite direction than one might expect. That is, subjects were significantly more likely to say that no one will know about the new hook in the Low Interest Condition (20%) as compared to the High Interest Condition (13%; $(\chi^2(1) = 5.41, p = .020$, two-tailed, $V_{Cramer} = .09, CI_{95\%}[.01, .17], n = 608)$. Moreover, since we found a significant difference between conditions, we conducted a logistic regression to see if answers to the knowledge question could fully explain our finding (as we indicated we would do in the preregistration document), and, in fact, they could not. Once knowledge is added into the model along with condition, there is still a highly significant effect of condition. See Fig. S5 for model comparison specifications and statistics.

As an even more conservative check, we can remove participants from our analysis who said that one or more people would find out about John using the new hook. When we do this, there is still a significant difference between the conditions in the proportion of subjects judging John's action morally acceptable with hardly any change in the effect size or confidence interval around the effect (Low Interest: 76%, High Interest: 42%; $(\chi^2(1) = 60.79, p < .001$, two-tailed, $V_{Cramer} = .35, CI_{95\%}[.26, .43], n = 507$.

D. Explanation Judgments: Materials. A different group of 200 subjects were recruited to make explanation judgments. The procedure for this part of the study was similar to that of Study 1, except that subjects read the High Interest fishing scenario used in Study 2a. 60 subjects were excluded for failing an attention check.

Subjects were presented with the scenario from the High Interest Condition, fishing context. Importantly, the characters in the story do not use explicit universalization reasoning as they do in the moral judgment scenario, above, which could bias subjects to choose universalization reasoning as the best explanation. Instead, subjects were randomly assigned to one of two conditions, which varied the reason that the fishermen chose to abstain from using the powerful fishing hook. In one condition, the fishermen simply say that using the new hooks is wrong (Wrong Condition), which captures the phenomenon of the fishermen being interested but abstaining from using the hooks for moral reasons without adding additional information. In the other condition, the fishermen say that they abstain from using the new hooks because of their allegiance to traditional fishing methods (Traditionalism Condition; this variation of the scenario is used in Study 2b). The two conditions yield similar patterns of results (see below). Only the results from the Wrong Condition are reported in the main text.

Subjects read the following introductory text: "On the following page, you will be asked to read a short story and answer a few simple questions about it. After the survey there will be an opportunity to tell us if something was confusing or unclear."

Subjects then read one of the following scenarios.

Traditionalism Condition Lake Wilson is a small lake in upstate New York. Each summer, a few dozen families move into small cottages near the lake for the season. The vacationers enjoy boating, swimming, and fishing in the lake and they've gotten to know each other over the course of many summers together.

Most people who like to fish in Lake Wilson catch a few fish each day and cook them for dinner. These people buy their fishing gear from a small tackle shop on the main road. Up until now, the tackle shop has sold one kind of fishing hook. But it has recently starting selling a new kind of hook that allows people to catch many more fish than they would with the older hook.

There are 20 people who regularly fish in Lake Wilson during the summer. If 5 or more people started using the new hook for fishing, then by the end of the summer, the entire fish population would collapse and there would be no more fish in Lake Wilson ever again. None of the vacationers would want that to happen. However, up to 3 people can use the new hooks with no decrease at all in the size and health of the fish population. Those people will be able to catch more fish more quickly, everyone else will still be able to catch the same amount of fish as before, and the fish population would continue to be exactly as large and as healthy as in past years.

It is impossible to tell what hook someone is using from a distance, so if someone starts using the new hooks, no one else will know about it. No one has bought the new hooks yet, however. John would like to use the new hooks because he would like to catch more fish faster. He is thinking about whether or not to buy the new hooks. John wants to find out what his neighbors think about the new hooks, so he decides he is going to visit the cottages of all the people who fish in Lake Wilson and ask them. John manages to talk to the other 19 people who regularly fish in the lake. When John sits down with each person to talk about the new hooks, each person says something like this: **"I would love to use those new hooks to catch more fish faster, but I think it is important to use traditional fishing methods, so I'm not going to use the new hook."** [Note: Bold used here for emphasis. Subjects did not see text bolded.]

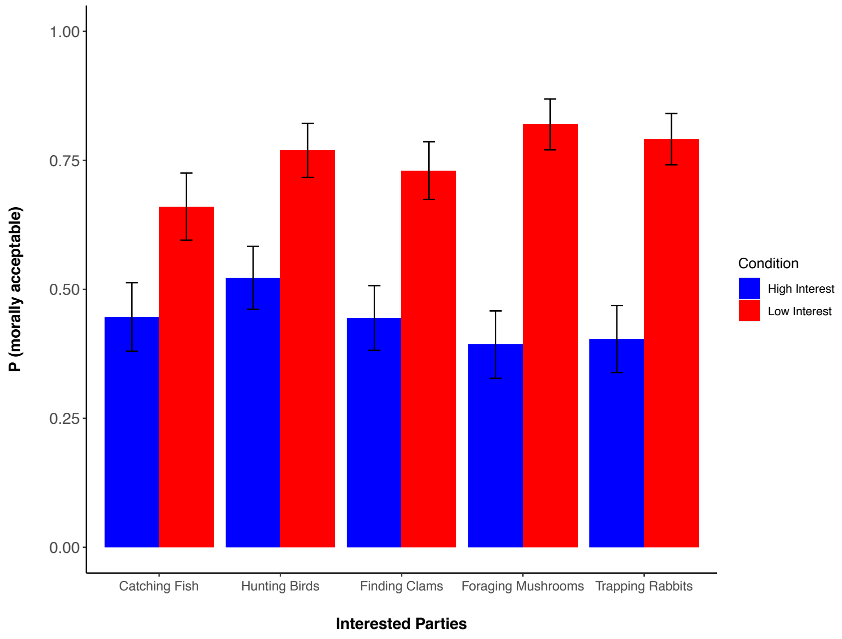

Fig. S3. Results of Study 2a, broken down by context.

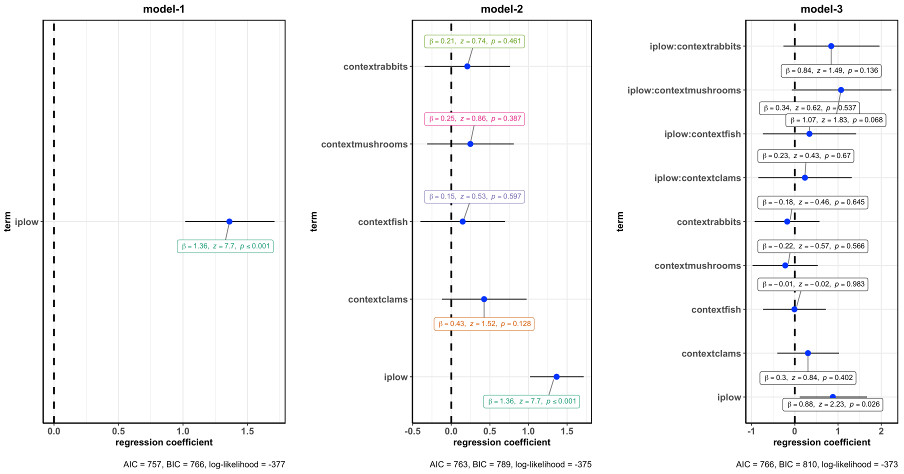

Fig. S4. Comparison of three possible models for Study 2a analysis. Model 1 includes only condition (High Interest/Low Interest) as a predictor. Model 2 includes context as well as condition. Model 3 includes the context x condition interaction. Model 1 fits the data best on AIC and BIC measures. Model 2 shows that there is no main effect of context. Model 3 shows that there is no significant interaction with context.

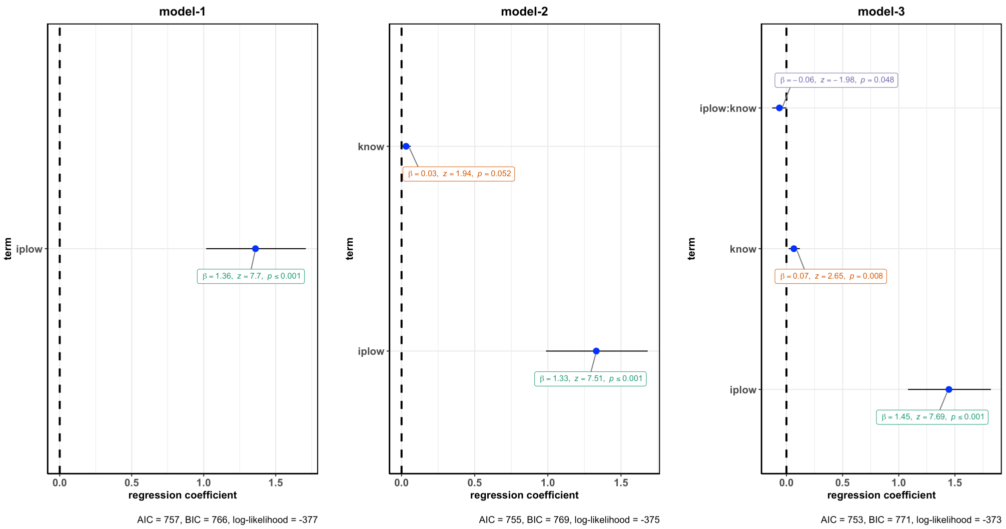

Fig. S5. Model comparisons for Study 2a. Can attributions of knowledge (the number of people who will know about John using the new hook) explain moral judgments? Knowledge attributions were entered into the logistic regression as integers. Three models are compared to predict moral permissibility judgments. The first model includes condition only. The second includes condition and knowledge. The third allows condition and knowledge to interact. The third model explains the data best, when considering AIC and BIC. Even when knowledge is entered into the model, there is still a large and significant impact of condition. Therefore, knowledge alone cannot entirely explain the findings.

John thinks to himself: No one else is going to use the new hooks. Under these circumstances, it's OK for me to use the new hooks and catch more fish.

Wrong Condition

Lake Wilson is a small lake in upstate New York. Each summer, a few dozen families move into small cottages near the lake for the season. The vacationers enjoy boating, swimming, and fishing in the lake and they've gotten to know each other over the course of many summers together.

Most people who like to fish in Lake Wilson catch a few fish each day and cook them for dinner. These people buy their fishing gear from a small tackle shop on the main road. Up until now, the tackle shop has sold one kind of fishing hook. But it has recently starting selling a new kind of hook that allows people to catch many more fish than they would with the older hook.

There are 20 people who regularly fish in Lake Wilson during the summer. If 5 or more people started using the new hook for fishing, then by the end of the summer, the entire fish population would collapse and there would be no more fish in Lake Wilson ever again. None of the vacationers would want that to happen. However, up to 3 people can use the new hooks with no decrease at all in the size and health of the fish population. Those people will be able to catch more fish more quickly, everyone else will still be able to catch the same amount of fish as before, and the fish population would continue to be exactly as large and as healthy as in past years.

It is impossible to tell what hook someone is using from a distance, so if someone starts using the new hooks, no one else will know about it. No one has bought the new hooks yet, however. John would like to use the new hooks because he would like to catch more fish faster. He is thinking about whether or not to buy the new hooks. John wants to find out what his neighbors think about the new hooks, so he decides he is going to visit the cottages of all the people who fish in Lake Wilson and ask them. John manages to talk to the other 19 people who regularly fish in the lake. When John sits down with each person to talk about the new hooks, each person says something like this: **"I would love to use those new hooks to catch more fish faster, but I think it's wrong to do that, so I'm not going to use the new hook." John thinks to himself: No one else is going to use the new hooks. Under these circumstances, it's OK for me to use the new hooks and catch more fish.** [Note: Bold used here for emphasis. Subjects did not see text bolded.]

Just like in Study 1, subjects were then asked to indicate whether each of four explanations were convincing explanations as to why that was wrong. These explanations were as follows:

1. Because that person could have helped more people.
2. Because that was unfair.
3. Because if everyone did that, the outcome would be bad.
4. Because that harmed someone.

(For the format of these questions, see Fig. S1)

Attention Check

At the end of the survey, subjects read the following: "Thanks for taking the time to do this survey. This final question is just here to confirm you are paying attention. Please do not answer this question (do not check any of the boxes). Instead, write 'I am paying attention' in the box labeled 'Other' below. Thanks so much for your help!" Subjects were presented with the following options: High School, Associate's Degree, Bachlor's Degree, Professional Degree or PhD, Some College. Subjects were excluded if they checked any of the levels of education or failed to write "I am paying attention" in the free-response box.

E. Explanation Judgments: Supplemental Results. **Wrong Condition** Participants explicitly endorse universalization as a good explanation for why John's behavior is wrong. Participants endorse universalization (86%) significantly more than harm (25%, $\chi^2(1) = 54.5, p < .001$), utility-maximization (24%, $\chi^2(1) = 56.8, p < .001$) or fairness (56%, $\chi^2(1) = 16.3, p < .001$). See Fig. S6.

Traditionalism Condition Participants explicitly endorse universalization as a good explanation for why John's behavior is wrong. Participants endorse universalization (83%) significantly more than harm (23%, $\chi^2(1) = 50.6.0, p < .001$), utility-maximization (19%, $\chi^2(1) = 57.9, p < .001$) or fairness (61%, $\chi^2(1) = 8.0, p < .005$). See Fig. S6.

Study 2b. This study was preregistered (https://aspredicted.org/blind.php?x=hd589d). We preregistered that we would stop data collection when 250 subjects passed the control questions. Therefore, 431 subjects participated in this study, recruited from Amazon MTURK through turkprime and were paid a small amount for their participation. 181 subjects were excluded for failing control questions, leaving 250 subjects included.

Subjects read the following instructions prior to beginning the study: "On the following pages you will be asked to read a short story and answer a few pages of questions about it. The story will remain the same from page to page; it is there for your reference. Only the questions will change. After the survey there will be an opportunity to let us know if something was confusing or unclear."

Subjects read the following stimuli. On each new page, the story appeared again for subjects' reference. For the first story only, we present all the questions asked to subjects. After that, we present only the text of the scenarios.

Threshold Condition

—-Page 1——

Lake Wilson is a small lake in upstate New York. Each summer, a few dozen families move into small cottages near the lake for the season. The vacationers enjoy boating, swimming, and fishing in the lake and they've gotten to know each other over the course of many summers together.

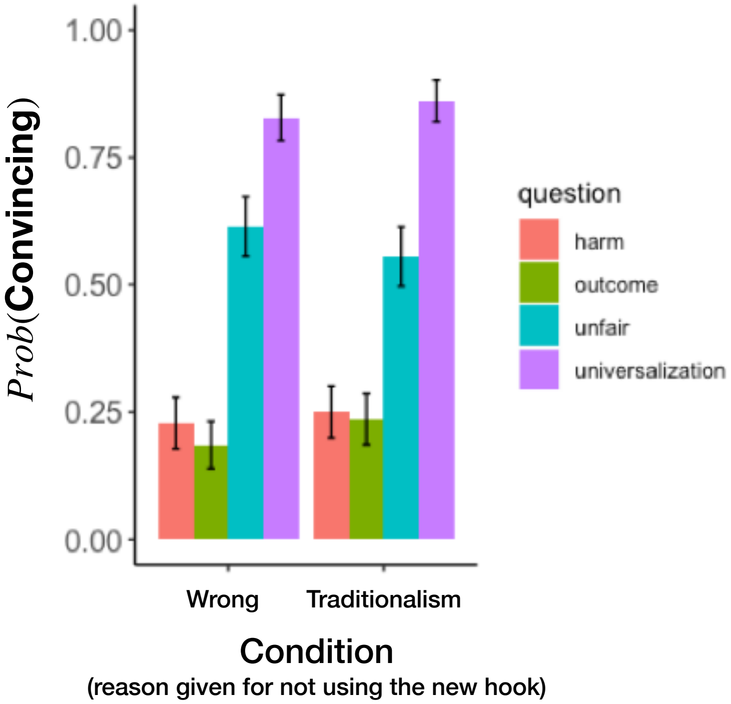

Fig. S6. Results of the explanation judgments from Study 2.

Most people who like to fish in Lake Wilson catch a few fish each day and cook them for dinner. These people buy their fishing gear from a small tackle shop on the main road. Up until now, the tackle shop has sold one kind of fishing hook. But it has recently starting selling a new kind of hook that allows people to catch many more fish than they would with the older hook.

There are 20 people who regularly fish in Lake Wilson during the summer. Only one vacationer, John, knows what will happen if people start using the new hooks. John knows that if 5 or more people started using the new hook for fishing, then by the end of the summer, the entire fish population would collapse and there would be no more fish in Lake Wilson ever again. None of the vacationers would want that to happen. However, up to 3 people can use the new hooks with no decrease at all in the size and health of the fish population. Those people will be able to catch more fish more quickly, everyone else will still be able to catch the same amount of fish as before, and the fish population would continue to be exactly as large and as healthy as in past years.

But, John knows that the rest of the vacationers assume nothing bad will happen if everyone starts using the new hook.

John wants to find out what his neighbors think about using the new hooks themselves, so he decides he is going to visit the cottages of all the people who fish in Lake Wilson and ask them. John manages to talk to the other 19 people who regularly fish in the lake. When John sits down with each person to talk about the new hooks, each person says something like this: "I would love to use those new hooks to catch more fish faster, but I feel committed to using traditional fishing methods, so I'm not going to use the new hook".

It is impossible to tell what hook someone is using from a distance, so if someone starts using the new hooks, no one else will know about it. No one has bought the new hooks yet. John would like to use the new hooks because he would like to catch more fish faster.

John thinks to himself: No one else is going to use the new hooks. Under these circumstances, it's OK for me to use the new hooks and catch more fish.

Please answer the following comprehension questions, so we know that you read the story.

If John starts using the new hooks, how many people will know about it? [Free Response.]

How many people, besides John, would like to catch more fish? [Free Response.]

How many people regularly fish in Lake Wilson in the summer? [Free Response.]

How many people, besides John, are actually going to use the new hooks? [Free Response.]

What do most of the vacationers (besides John) think will happen if everyone starts using the new hooks? [The fish population will collapse/Nothing bad will happen to the fish population]

Note: In the No Threshold Condition, this question reads: What do the vacationers think will happen if everyone starts using the new hooks? [The fish population will collapse/Nothing bad will happen to the fish population]

–Page 2–

Is it morally acceptable for John to use the new hooks? [Yes/No]

–Page 3–

Is there a rule on the lake that the new hooks are forbidden? [Yes/No]

–Page 4–

Will it make a difference to the fish population if John uses the new hooks? [It will make the fish population more healthy/It will make the fish population less healthy/It will not make a difference to the fish population]

No Threshold Condition

Lake Wilson is a small lake in upstate New York. Each summer, a few dozen families move into small cottages near the lake for the season. The vacationers enjoy boating, swimming, and fishing in the lake and they've gotten to know each other over the course of many summers together.

Most people who like to fish in Lake Wilson catch a few fish each day and cook them for dinner. These people buy their fishing gear from a small tackle shop on the main road. Up until now, the tackle shop has sold one kind of fishing hook. But it has recently starting selling a new kind of hook that allows people to catch many more fish than they would with the older hook.

There are 20 people who regularly fish in Lake Wilson during the summer. John knows that all of them can use the new hooks with no decrease at all in the size and health of the fish population. And, John knows that the rest of the vacationers also assume nothing bad will happen if everyone starts using the hook.

John wants to find out what his neighbors think about using the new hooks themselves, so he decides he is going to visit the cottages of all the people who fish in Lake Wilson and ask them. John manages to talk to the other 19 people who regularly fish in the lake. When John sits down with each person to talk about the new hooks, each person says something like this: "I would love to use those new hooks to catch more fish faster, but I feel committed to using traditional fishing methods, so I'm not going to use the new hook".

It is impossible to tell what hook someone is using from a distance, so if someone starts using the new hooks, no one else will know about it. No one has bought the new hooks yet. John would like to use the new hooks because he would like to catch more fish faster.

John thinks to himself: No one else is going to use the new hooks. Under these circumstances, it's OK for me to use the new hooks and catch more fish.

Exclusion Criteria: 1. How many people, besides John, are interested in catching more fish? To be included in the study, participants must report the value given for IP in the story (19).

2. How many people regularly fish in Lake Wilson in the summer? To be included, subjects must answer 19, 20, or 21.

3. How many people, besides John, are actually going to use the new hooks? To be included, subjects must answer 0.

4. Will it make a difference to the fish population if John uses the new hooks? There are three possible answers to this question: (A) It will make the fish population more healthy (B) It will make the fish population less healthy (C) It will not make a difference to the fish population. To be included, subjects must answer (C).

5. What do most of the vacationers (besides John) think will happen if everyone starts using the new hooks? [In the No Threshold Condition: What do the vacationers think will happen if everyone starts using the new hooks?] There are two possible answers to this question: (A) The fish population will collapse (B) Nothing bad will happen to the fish population. Subjects must answer (B) to be included.

Study 2b: Conceptual Replication 1. This study was preregistered (see http://aspredicted.org/blind.php?x=9mn2cf). 350 subjects participated in this study, recruited from Amazon MTURK through turkprime and were paid a small amount for their participation. 140 subjects were excluded for failing control questions.

Subjects read the following instructions prior to beginning the study: "On the following pages you will be asked to read a short story and answer a few pages of questions about it. The story will remain the same from page to page; it is there for your reference. Only the questions will change. After the survey there will be an opportunity to let us know if something was confusing or unclear."

Subjects read the following stimuli. On each new page, the story appeared again for subjects' reference. For the first story only, we present all the questions asked to subjects. After that, we present only the text of the scenarios.

Threshold Condition

—-Page 1——

Lake Wilson is a small lake in upstate New York. Each summer, a few dozen families move into small cottages near the lake for the season. The vacationers enjoy boating, swimming, and fishing in the lake and they've gotten to know each other over the course of many summers together. Most people who like to fish in Lake Wilson catch a few fish each day and cook them for dinner. These people buy their fishing gear from a small tackle shop on the main road. Up until now, the tackle shop has sold one kind of fishing hook. But it has recently starting selling a new kind of hook that allows people to catch many more fish than they would with the older hook.

There are 20 people who regularly fish in Lake Wilson during the summer. If 5 or more people started using the new hook for fishing, then by the end of the summer, the entire fish population would collapse and there would be no more fish in Lake Wilson ever again. None of the vacationers would want that to happen. However, up to 3 people can use the new hooks with no decrease at all in the size and health of the fish population. Those people will be able to catch more fish more quickly, everyone else will still be able to catch the same amount of fish as before, and the fish population would continue to be exactly as large and as healthy as in past years.

It is impossible to tell what hook someone is using from a distance, so if someone starts using the new hooks, no one else will know about it. No one has bought the new hooks yet, however. John would like to use the new hooks because he would like to catch more fish faster. He is thinking about whether or not to buy the new hooks. John wants to find out what his neighbors think about the new hooks, so he decides he is going to visit the cottages of all the people who fish in Lake Wilson and ask them. John manages to talk to the other 19 people who regularly fish in the lake. When John sits down with each person to talk about the new hooks, each person says something like this: "I would love to use those new hooks to catch more fish faster, but I think it is important to use traditional fishing methods, so I'm not going to use the new hook."

John thinks to himself: No one else is going to use the new hooks. Under these circumstances, it's OK for me to use the new hooks and catch more fish.

Please answer the following comprehension questions, so we know that you read the story.

If John starts using the new hooks, how many people will know about it? [Free Response.]

How many people, besides John, would like to catch more fish? [Free Response.]

How many people regularly fish in Lake Wilson in the summer? [Free Response.]

How many people, besides John, are actually going to use the new hooks? [Free Response.]

–Page 2–

Is it morally acceptable for John to use the new hooks? [Yes/No]

–Page 3–

Is there a rule on the lake that the new hooks are forbidden? [Yes/No]

–Page 4–

Will it make a difference to the fish population if John uses the new hooks? [It will make the fish population more healthy/It will make the fish population less healthy/It will not make a difference to the fish population]

No Threshold Condition

Lake Wilson is a small lake in upstate New York. Each summer, a few dozen families move into small cottages near the lake for the season. The vacationers enjoy boating, swimming, and fishing in the lake and they've gotten to know each other over the course of many summers together. Most people who like to fish in Lake Wilson catch a few fish each day and cook them for dinner. These people buy their fishing gear from a small tackle shop on the main road. Up until now, the tackle shop has sold one kind of fishing hook. But it has recently starting selling a new kind of hook that allows people to catch many more fish than they would with the older hook. There are 20 people who regularly fish in Lake Wilson during the summer. All of them can use the new hooks with no decrease at all in the size and health of the fish population.

It is impossible to tell what hook someone is using from a distance, so if someone starts using the new hooks, no one else will know about it. No one has bought the new hooks yet, however. John would like to use the new hooks because he would like to catch more fish faster. He is thinking about whether or not to buy the new hooks. John wants to find out what his neighbors think about the new hooks, so he decides he is going to visit the cottages of all the people who fish in Lake Wilson and ask them. John manages to talk to the other 19 people who regularly fish in the lake. When John sits down with each person to talk about the new hooks, each person says something like this: "I would love to use those new hooks to catch more fish faster, but I think it is important to use traditional fishing methods, so I'm not going to use the new hook."

John thinks to himself: No one else is going to use the new hooks. Under these circumstances, it's OK for me to use the new hooks and catch more fish.

Exclusion Criteria: 1. How many people, besides John, are interested in catching more fish? To be included in the study, participants must report the value given for IP in the story (19).

2. How many people regularly fish in Lake Wilson in the summer? To be included, subjects must answer 19, 20, or 21.

3. How many people, besides John, are actually going to use the new hooks? To be included, subjects must answer 0.

4. Will it make a difference to the fish population if John uses the new hooks? There are three possible answers to this question: (A) It will make the fish population more healthy (B) It will make the fish population less healthy (C) It will not make a difference to the fish population. To be included, subjects must answer (C).

F. Results. As predicted by the universalization model, more participants judged the "no threshold" case permissible (91%) than judged the "threshold case" permissible (56%, ($\chi^2(1) = 34.4, p < .001$, two-tailed, $V_{Cramer} = .40, CI_{95\%}[.31, .52], n = 210$,see Fig. S7).

Study 2b: Conceptual Replication 2. This study was preregistered (http://aspredicted.org/blind.php?x=5iy565). 350 subjects participated in this study, recruited from Amazon MTURK through turkprime and were paid a small amount for their participation. 137 subjects were excluded for failing control questions. This study was identical to the original Study 2b (described above) except that the reason that the fishermen give for abstaining from using the new fishing hook is different. They say that they find using the new hook wrong, rather than abstaining from it due to their adherence to traditional fishing practices. See below for precise text of stimuli. The questions asked of subjects and the exclusion criteria are identical to those above. Only the text of the scenarios are given here.

Threshold Condition

Lake Wilson is a small lake in upstate New York. Each summer, a few dozen families move into small cottages near the lake for the season. The vacationers enjoy boating, swimming, and fishing in the lake and they've gotten to know each other over the course of many summers together. Most people who like to fish in Lake Wilson catch a few fish each day and cook them for dinner. These people buy their fishing gear from a small tackle shop on the main road. Up until now, the tackle shop has sold one kind of fishing hook. But it has recently starting selling a new kind of hook that allows people to catch many more fish than they would with the older hook.

There are 20 people who regularly fish in Lake Wilson during the summer. If 5 or more people started using the new hook for fishing, then by the end of the summer, the entire fish population would collapse and there would be no more fish in Lake Wilson ever again. None of the vacationers would want that to happen. However, up to 3 people can use the new hooks with no decrease at all in the size and health of the fish population. Those people will be able to catch more fish more quickly, everyone else will still be able to catch the same amount of fish as before, and the fish population would continue to be exactly as large and as healthy as in past years.

It is impossible to tell what hook someone is using from a distance, so if someone starts using the new hooks, no one else will know about it. No one has bought the new hooks yet, however. John would like to use the new hooks because he would like to catch more fish faster. He is thinking about whether or not to buy the new hooks. John wants to find out what his neighbors think about the new hooks, so he decides he is going to visit the cottages of all the people who fish in Lake Wilson and ask them. John manages to talk to the other 19 people who regularly fish in the lake. When John sits down with each person to talk about the new hooks, each person says something like this: "I would love to use those new hooks to catch more fish faster, but I think using them is wrong. So I'm not going to use the new hooks."

John thinks to himself: No one else is going to use the new hooks. Under these circumstances, it's OK for me to use the new hooks and catch more fish.

No Threshold Condition

Lake Wilson is a small lake in upstate New York. Each summer, a few dozen families move into small cottages near the lake for the season. The vacationers enjoy boating, swimming, and fishing in the lake and they've gotten to know each other over the course of many summers together. Most people who like to fish in Lake Wilson catch a few fish each day and cook them for dinner. These people buy their fishing gear from a small tackle shop on the main road. Up until now, the tackle shop has sold one kind of fishing hook. But it has recently starting selling a new kind of hook that allows people to catch many more fish than they would with the older hook. There are 20 people who regularly fish in Lake Wilson during the summer. All of them can use the new hooks with no decrease at all in the size and health of the fish population.

It is impossible to tell what hook someone is using from a distance, so if someone starts using the new hooks, no one else will know about it. No one has bought the new hooks yet, however. John would like to use the new hooks because he would like to catch more fish faster. He is thinking about whether or not to buy the new hooks. John wants to find out what his neighbors think about the new hooks, so he decides he is going to visit the cottages of all the people who fish in Lake Wilson

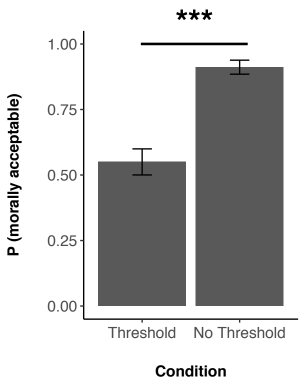

Fig. S7. Results from Study 2b: Conceptual Replication 1. There is a significant difference between the Threshold and No Threshold Conditions, as predicted by universalization (but not by a norms-based model). Error bars are standard error of the mean. *** indicates $p < 0.001$

and ask them. John manages to talk to the other 19 people who regularly fish in the lake. When John sits down with each person to talk about the new hooks, each person says something like this: "I would love to use those new hooks to catch more fish faster, but I think using them is wrong. So I'm not going to use the new hooks."

John thinks to himself: No one else is going to use the new hooks. Under these circumstances, it's OK for me to use the new hooks and catch more fish.

G. Results. As predicted by the universalization model, and replicating the effect reported in the original Study 2b, more participants judged the "no threshold" case permissible (83%) than judged the "threshold case" permissible (45%, ($\chi^2(1) = 33.35, p < .001$, two-tailed, $V_{Cramer} = .40, CI_{95\%}[.28, .52], n = 213$,see Fig. S8).

Study 3

Materials. 1242 subjects participated in this study, recruited from Amazon MTURK through turkprime and were paid a small amount for their participation. Subjects were divided into two groups: the Moral Judgment Group ($n = 840$) and the Expected Utility Group ($n = 402$; 2 additional subjects were accidentally allowed to take the experiment after our 400 subject cap). Subjects in both groups were randomly assigned to one context (Fisherman or Tour Boat) and one condition (High Interest or Low Interest). Subjects in the Moral Judgment Group answered different questions about the scenarios than did subjects in the Expected Utility Group.

Moral Judgment Group

Exclusion Criteria for the Moral Judgment Group: 1. How many people, besides John, would like to use the new motor oil if there were no bad effects of doing so? To be included in the study, participants in the High Interest Condition must answer 19 and subjects in the Low Interest Condition must answer 0.

2. How many boats operate on Lake Wilson? To be included, subjects must answer 20.

3. How many people, besides John, are actually going to use the new motor oil? To be included, subjects must answer 0.

4. Will it make a difference to the fish population if John uses the new motor oil? There are three possible answers to this question: (A) It will make the fish population more healthy (B) It will make the fish population less healthy (C) It will not make a difference to the fish population. To be included, subjects must answer (C).

Exclusions: 284 subjects were excluded from the study for failing one or more control questions.

Subjects read the following instructions prior to beginning the study: "On the following pages you will be asked to read a short story and answer questions about it. The questions on each page of the survey will be different, but the story will remain the same. (The story will appear on each page for your reference.) After the survey there will be an opportunity to let us know if something was confusing or unclear."

Subjects read the following stimuli. On each new page, the story appeared again for subjects' reference.

Condition: High Interest

Context: Tour Boat

–Page 1–

Lake Wilson is a small lake in upstate New York. Each summer, 20 people make their livelihood on the water. All but one of these people makes their livelihood by catching fish and selling them. John makes his livelihood a different way: By giving tours of the lake to tourists on his boat.

Everybody who makes their livelihood on the water uses a motor boat. The motor boats on Lake Wilson move around the lake relatively slowly, mostly because the engines use a crude form of motor oil. Up until now, the boat shop in town has sold only one kind of motor oil. But recently it started selling a new kind of motor oil that allows the boats to move much more quickly.

This would allow everyone who makes their livelihood on the lake to save time. All the fishermen would be able to move around the lake faster and thereby catch the number of fish they need each day in less time. John would also save time because the people who take his tours want to see three sites: the coral reef, the waterfall, and the lake's island. With the new motor oil, John can show everyone all three sites in less time.

The new motor oil also releases a compound, BetaX, into the water. Small amounts of BetaX in the water do not effect the fish. But, if BetaX levels start to exceed 30 parts per million, the fish will die. If the fish die, John will still be able to give just as many tours on Lake Wilson, but the people that make their livelihood by catching fish will have to find different jobs.

There are 20 total boaters who operate boats on Lake Wilson: John with his tour boat, and 19 fishermen with their fishing boats. If 7 or more boaters started using the new motor oil, then by the end of the summer all the fish would be dead. However, up to 3 boaters can use the new motor oil with no decrease at all in the size and health of the fish population. Those boaters will be able to drive around the lake faster, everybody else will drive around at the same speed as before, and the fish population would continue to be exactly as large and as healthy as in past years.

It is impossible to tell what motor oil someone is using, so if somebody starts using the new motor oil, no one else will know about it. No one has bought the new motor oil yet, however. John would like to use the new motor oil because he would like the ability to drive around the lake more quickly. He is thinking about whether or not to buy the new motor oil. John wants to find out what the other people who make their livelihood on the water think, so he decides he is going to visit the cottages of all the people who fish in Lake Wilson and ask them. John manages to talk to the 19 people who regularly fish in the lake. When John sits down with each person to talk about the new motor oil, every one of them says something like this: "I would love to use that new motor oil to move around on the lake more quickly, but what would happen if everyone did that?

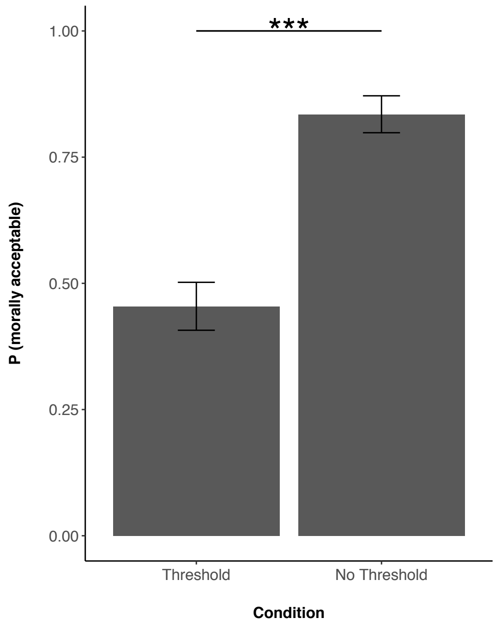

Fig. S8. Results from Study 2b: Conceptual Replication 2. There is a significant difference between the Threshold and No Threshold Conditions, as predicted by universalization (but not by a norms-based model). Error bars are standard error of the mean. *** indicates $p < 0.001$

If everyone used the new motor oil, all the fish would disappear from the lake. I don't want that to happen, so I'm not going to use the new motor oil."

John thinks to himself: No one else is going to use the new motor oil. Under these circumstances, it's OK for me to use the new motor oil and drive around the lake more quickly.

Please answer the following comprehension questions, so we know that you read the story.

If John starts using the new motor oil, how many people will know about it? [Free Response.]

How many people, besides John, would like to use the new motor oil if there were no bad effects of doing so? [Free Response.]

How many boats operate on Lake Wilson? [Free Response.]

How many people, besides John, are actually going to use the new motor oil? [Free Response.]

–Page 2–

Is it morally acceptable for John to use the new motor oil? [Yes/No]

–Page 3–

Is there a rule on the lake that the new motor oil is forbidden? [Yes/No]

–Page 4–

Will it make a difference to the fish population if John uses the new motor oil? [It will make the fish population more healthy/It will make the fish population less healthy/It will not make a difference to the fish population]

Condition: Low Interest

Context: Tour Boat

Lake Wilson is a small lake in upstate New York. Each summer, 20 people make their livelihood on the water. All but one of these people makes their livelihood by catching fish and selling them. John makes his livelihood a different way: By giving tours of the lake to tourists on his boat.

Everybody who makes their livelihood on the water uses a motor boat. The motor boats on Lake Wilson move around the lake relatively slowly, mostly because the engines use a crude form of motor oil. Up until now, the boat shop in town has sold only one kind of motor oil. But recently it started selling a new kind of motor oil that allows the boats to move much more quickly.

This would allow everyone who makes their livelihood on the lake to save time. All the fishermen would be able to move around the lake faster and thereby catch the number of fish they need each day in less time. John would also save time because the people who take his tours want to see three sites: the coral reef, the waterfall, and the lake's island. With the new motor oil, John can show everyone all three sites in less time.

The new motor oil also releases a compound, BetaX, into the water. Small amounts of BetaX in the water do not effect the fish. But, if BetaX levels start to exceed 30 parts per million, the fish will die. If the fish die, John will still be able to give just as many tours on Lake Wilson, but the people that make their livelihood by catching fish will have to find different jobs.

There are 20 total boaters who operate boats on Lake Wilson: John with his tour boat, and 19 fishermen with their fishing boats. If 7 or more boaters started using the new motor oil, then by the end of the summer all the fish would be dead. However, up to 3 boaters can use the new motor oil with no decrease at all in the size and health of the fish population. Those boaters will be able to drive around the lake faster, everybody else will drive around at the same speed as before, and the fish population would continue to be exactly as large and as healthy as in past years.

It is impossible to tell what motor oil someone is using, so if somebody starts using the new motor oil, no one else will know about it. No one has bought the new motor oil yet, however. John would like to use the new motor oil because he would like the ability to drive around the lake more quickly. He is thinking about whether or not to buy the new motor oil. John wants to find out what the other people who make their livelihood on the water think, so he decides he is going to visit the cottages of all the people who fish in Lake Wilson and ask them. John manages to talk to the 19 people who regularly fish in the lake. When John sits down with each person to talk about the new motor oil, every one of them says something like this: "I'm just not interested in using that new motor oil. I really only want to move my boat slowly. I like going at a leisurely pace and spending all day on the lake. I'm not in any rush and I don't need to speed anything up. Besides, if everyone used the new motor oil, all the fish would disappear from the lake. I don't want that to happen. But even if there was no BetaX released into the lake by the new motor oil, I wouldn't want to use it anyway."

John thinks to himself: No one else is going to use the new motor oil. Under these circumstances, it's OK for me to use the new motor oil and drive around the lake more quickly.

Condition: High Interest

Context: Fisherman

Lake Wilson is a small lake in upstate New York. Each summer, 20 people make their livelihood on the water. All but one of these people makes their livelihood by catching fish and selling them. Steve makes his livelihood a different way: By giving tours of the lake to tourists on his boat.

Everybody who makes their livelihood on the water uses a motor boat. The motor boats on Lake Wilson move around the lake relatively slowly, mostly because the engines use a crude form of motor oil. Up until now, the boat shop in town has sold only one kind of motor oil. But recently it started selling a new kind of motor oil that allows the boats to move much more quickly.

This would allow everyone who makes their livelihood on the lake to save time. All the fishermen would be able to move around the lake faster and thereby catch the number of fish they need each day in less time. Steve would also save time because

the people who take his tours want to see three sites: the coral reef, the waterfall, and the lake's island. With the new motor oil, Steve can show everyone all three sites in less time.

The new motor oil also releases a compound, BetaX, into the water. Small amounts of BetaX in the water do not effect the fish. But, if BetaX levels start to exceed 30 parts per million, the fish will die. If the fish die, Steve will still be able to give just as many tours on Lake Wilson, but the people that make their livelihood by catching fish will have to find different jobs.

There are 20 total boaters who operate boats on Lake Wilson: Steve with his tour boat, and 19 fishermen with their fishing boats. If 7 or more boaters started using the new motor oil, then by the end of the summer all the fish would be dead. However, up to 3 boaters can use the new motor oil with no decrease at all in the size and health of the fish population. Those boaters will be able to drive around the lake faster, everybody else will drive around at the same speed as before, and the fish population would continue to be exactly as large and as healthy as in past years.

It is impossible to tell what motor oil someone is using, so if somebody starts using the new motor oil, no one else will know about it. No one has bought the new motor oil yet, however. John is one of the people who operates a fishing boat. He would like to use the new motor oil because he would like the ability to drive around the lake more quickly. He is thinking about whether or not to buy the new motor oil. John wants to find out what the other people who make their livelihood on the water think, so he decides he is going to visit the cottages of all the people who work on Lake Wilson and ask them. John manages to talk to other 19 people who operate boats on the lake (Steve and the 18 other fishermen). When John sits down with each person to talk about the new motor oil, every one of them says something like this: "I would love to use that new motor oil to move around on the lake more quickly, but what would happen if everyone did that? If everyone used the new motor oil, all the fish would disappear from the lake. I don't want that to happen, so I'm not going to use the new motor oil."

John thinks to himself: No one else is going to use the new motor oil. Under these circumstances, it's OK for me to use the new motor oil and drive around the lake more quickly.

Condition: Low Interest

Context: Fisherman

Lake Wilson is a small lake in upstate New York. Each summer, 20 people make their livelihood on the water. All but one of these people makes their livelihood by catching fish and selling them. Steve makes his livelihood a different way: By giving tours of the lake to tourists on his boat.

Everybody who makes their livelihood on the water uses a motor boat. The motor boats on Lake Wilson move around the lake relatively slowly, mostly because the engines use a crude form of motor oil. Up until now, the boat shop in town has sold only one kind of motor oil. But recently it started selling a new kind of motor oil that allows the boats to move much more quickly.

This would allow everyone who makes their livelihood on the lake to save time. All the fishermen would be able to move around the lake faster and thereby catch the number of fish they need each day in less time. Steve would also save time because the people who take his tours want to see three sites: the coral reef, the waterfall, and the lake's island. With the new motor oil, Steve can show everyone all three sites in less time.

The new motor oil also releases a compound, BetaX, into the water. Small amounts of BetaX in the water do not effect the fish. But, if BetaX levels start to exceed 30 parts per million, the fish will die. If the fish die, Steve will still be able to give just as many tours on Lake Wilson, but the people that make their livelihood by catching fish will have to find different jobs.

There are 20 total boaters who operate boats on Lake Wilson: Steve with his tour boat, and 19 fishermen with their fishing boats. If 7 or more boaters started using the new motor oil, then by the end of the summer all the fish would be dead. However, up to 3 boaters can use the new motor oil with no decrease at all in the size and health of the fish population. Those boaters will be able to drive around the lake faster, everybody else will drive around at the same speed as before, and the fish population would continue to be exactly as large and as healthy as in past years.

It is impossible to tell what motor oil someone is using, so if somebody starts using the new motor oil, no one else will know about it. No one has bought the new motor oil yet, however. John is one of the people who operates a fishing boat. He would like to use the new motor oil because he would like the ability to drive around the lake more quickly. He is thinking about whether or not to buy the new motor oil. John wants to find out what the other people who make their livelihood on the water think, so he decides he is going to visit the cottages of all the people who work on Lake Wilson and ask them. John manages to talk to other 19 people who operate boats on the lake (Steve and the 18 other fishermen). When John sits down with each person to talk about the new motor oil, every one of them says something like this: "I'm just not interested in using that new motor oil. I really only want to move my boat slowly. I like going at a leisurely pace and spending all day on the lake. I'm not in any rush and I don't need to speed anything up. Besides, if everyone used the new motor oil, all the fish would disappear from the lake. I don't want that to happen. But even if there was no BetaX released into the lake by the new motor oil, I wouldn't want to use it anyway."

John thinks to himself: No one else is going to use the new motor oil. Under these circumstances, it's OK for me to use the new motor oil and drive around the lake more quickly.

Expected Utility Group

Exclusion Criteria for the Expected Utility Group: How boats operate on Lake Wilson? To be included, subjects must answer 20.

Exclusions: 16 subjects were excluded for failing the control question.

Subjects read the following instructions prior to beginning the study: "On the following pages you will be asked to read a short story and answer questions about it. The questions on each page of the survey will be different, but the story will remain

the same. (The story will appear on each page for your reference.) After the survey there will be an opportunity to let us know if something was confusing or unclear."

Subjects read the following stimuli. On each new page, the story appeared again for subjects' reference.

Condition: High Interest

Context: Tour Boat

Lake Wilson is a small lake in upstate New York. Each summer, 20 people make their livelihood on the water. All but one of these people makes their livelihood by catching fish and selling them. John makes his livelihood a different way: By giving tours of the lake to tourists on his boat.

Everybody who makes their livelihood on the water uses a motor boat. The motor boats on Lake Wilson move around the lake relatively slowly, mostly because the engines use a crude form of motor oil. Up until now, the boat shop in town has sold only one kind of motor oil. But recently it started selling a new kind of motor oil that allows the boats to move much more quickly.

This would allow everyone who makes their livelihood on the lake to save time. All the fishermen would be able to move around the lake faster and thereby catch the number of fish they need each day in less time. John would also save time because the people who take his tours want to see three sites: the coral reef, the waterfall, and the lake's island. With the new motor oil, John can show everyone all three sites in less time.

The new motor oil also releases a compound, BetaX, into the water. Small amounts of BetaX in the water do not effect the fish. But, if BetaX levels start to exceed 30 parts per million, the fish will die. If the fish die, John will still be able to give just as many tours on Lake Wilson, but the people that make their livelihood by catching fish will have to find different jobs.

There are 20 total boaters who operate boats on Lake Wilson: John with his tour boat, and 19 fishermen with their fishing boats. If 7 or more boaters started using the new motor oil, then by the end of the summer all the fish would be dead. However, up to 3 boaters can use the new motor oil with no decrease at all in the size and health of the fish population. Those boaters will be able to drive around the lake faster, everybody else will drive around at the same speed as before, and the fish population would continue to be exactly as large and as healthy as in past years.

Now suppose that:

1. John decides to use the new motor oil
2. All of the fishermen also decide to use the new motor oil

Please answer the following questions.

Would John be better off, worse off, or the same as he is right now? [Better off / Worse off / The same]

Would the fishermen be better off, worse off, or the same as they are right now? [Better off / Worse off / The same]

How likely is it that John will save time and still be able to give as many tours as before? [More likely than before / Less likely than before / The same as before]

How many boats operate on Lake Wilson? [Free Response]

Condition: Low Interest

Context: Tour Boat

Lake Wilson is a small lake in upstate New York. Each summer, 20 people make their livelihood on the water. All but one of these people makes their livelihood by catching fish and selling them. John makes his livelihood a different way: By giving tours of the lake to tourists on his boat.

Everybody who makes their livelihood on the water uses a motor boat. The motor boats on Lake Wilson move around the lake relatively slowly, mostly because the engines use a crude form of motor oil. Up until now, the boat shop in town has sold only one kind of motor oil. But recently it started selling a new kind of motor oil that allows the boats to move much more quickly.

This would allow everyone who makes their livelihood on the lake to save time. All the fishermen would be able to move around the lake faster and thereby catch the number of fish they need each day in less time. John would also save time because the people who take his tours want to see three sites: the coral reef, the waterfall, and the lake's island. With the new motor oil, John can show everyone all three sites in less time.

The new motor oil also releases a compound, BetaX, into the water. Small amounts of BetaX in the water do not effect the fish. But, if BetaX levels start to exceed 30 parts per million, the fish will die. If the fish die, John will still be able to give just as many tours on Lake Wilson, but the people that make their livelihood by catching fish will have to find different jobs.

There are 20 total boaters who operate boats on Lake Wilson: John with his tour boat, and 19 fishermen with their fishing boats. If 7 or more boaters started using the new motor oil, then by the end of the summer all the fish would be dead. However, up to 3 boaters can use the new motor oil with no decrease at all in the size and health of the fish population. Those boaters will be able to drive around the lake faster, everybody else will drive around at the same speed as before, and the fish population would continue to be exactly as large and as healthy as in past years.

Now suppose that: 1. John decides to use the new motor oil 2. None of the fishermen decide to use the new motor oil

Please answer the following questions.

Condition: High Interest

Context: Fisherman

Lake Wilson is a small lake in upstate New York. Each summer, 20 people make their livelihood on the water. All but one of these people makes their livelihood by catching fish and selling them. Steve makes his livelihood a different way: By giving tours of the lake to tourists on his boat.

Everybody who makes their livelihood on the water uses a motor boat. The motor boats on Lake Wilson move around the lake relatively slowly, mostly because the engines use a crude form of motor oil. Up until now, the boat shop in town has sold only one kind of motor oil. But recently it started selling a new kind of motor oil that allows the boats to move much more quickly.

This would allow everyone who makes their livelihood on the lake to save time. All the fishermen would be able to move around the lake faster and thereby catch the number of fish they need each day in less time. Steve would also save time because the people who take his tours want to see three sites: the coral reef, the waterfall, and the lake's island. With the new motor oil, Steve can show everyone all three sites in less time.

The new motor oil also releases a compound, BetaX, into the water. Small amounts of BetaX in the water do not effect the fish. But, if BetaX levels start to exceed 30 parts per million, the fish will die. If the fish die, Steve will still be able to give just as many tours on Lake Wilson, but the people that make their livelihood by catching fish will have to find different jobs.

There are 20 total boaters who operate boats on Lake Wilson: Steve with his tour boat, and 19 fishermen with their fishing boats. If 7 or more boaters started using the new motor oil, then by the end of the summer all the fish would be dead. However, up to 3 boaters can use the new motor oil with no decrease at all in the size and health of the fish population. Those boaters will be able to drive around the lake faster, everybody else will drive around at the same speed as before, and the fish population would continue to be exactly as large and as healthy as in past years.

John is one of the fishermen who operates a boat on Lake Wilson.

Now suppose that: 1. John decides to use the new motor oil 2. All of the other boaters also decide to use the new motor oil

Please answer the following questions.

Condition: Low Interest

Context: Fisherman

Lake Wilson is a small lake in upstate New York. Each summer, 20 people make their livelihood on the water. All but one of these people makes their livelihood by catching fish and selling them. Steve makes his livelihood a different way: By giving tours of the lake to tourists on his boat.

Everybody who makes their livelihood on the water uses a motor boat. The motor boats on Lake Wilson move around the lake relatively slowly, mostly because the engines use a crude form of motor oil. Up until now, the boat shop in town has sold only one kind of motor oil. But recently it started selling a new kind of motor oil that allows the boats to move much more quickly.

This would allow everyone who makes their livelihood on the lake to save time. All the fishermen would be able to move around the lake faster and thereby catch the number of fish they need each day in less time. Steve would also save time because the people who take his tours want to see three sites: the coral reef, the waterfall, and the lake's island. With the new motor oil, Steve can show everyone all three sites in less time.

The new motor oil also releases a compound, BetaX, into the water. Small amounts of BetaX in the water do not effect the fish. But, if BetaX levels start to exceed 30 parts per million, the fish will die. If the fish die, Steve will still be able to give just as many tours on Lake Wilson, but the people that make their livelihood by catching fish will have to find different jobs.

There are 20 total boaters who operate boats on Lake Wilson: Steve with his tour boat, and 19 fishermen with their fishing boats. If 7 or more boaters started using the new motor oil, then by the end of the summer all the fish would be dead. However, up to 3 boaters can use the new motor oil with no decrease at all in the size and health of the fish population. Those boaters will be able to drive around the lake faster, everybody else will drive around at the same speed as before, and the fish population would continue to be exactly as large and as healthy as in past years.

John is one of the fishermen who operates a boat on Lake Wilson.

Now suppose that: 1. John decides to use the new motor oil 2. None of the other boaters decide to use the new motor oil

Please answer the following questions.

Supplemental Results/Statistical Details. In the main text, we report a collapsed version of the results from the expected utility group. Here, in Table S1 we report the full results.

Study 4a

Materials. This study was preregistered (see https://aspredicted.org/blind.php?x=c44jr2). 700 subjects participated in this study, recruited from Amazon MTURK through turkprime and were paid a small amount for their participation. Subjects were randomly assigned to 1 of 2 conditions. 4,7 Condition: Up to 4 people can use the new hook with no effect on the fish population; once 7 people use the new hook the fish population will go extinct. 10,13 Condition: Up to 10 people can use the new hook with no effect on the fish population; once 13 people use the new hook the fish population will go extinct.

Each subject was told that N people are interested in using the new hook. Subjects answered a series of questions about the story. Subjects then read the same story, the only change being that a new value of N was given. N was chosen at random without replacement from the following values until all values of N were seen by each subject: 0,2,7,8,13,19.

Pre-registered Exclusion Criteria:

1. How many people, besides John, would like to use the new hooks if there were no bad effects of doing so? To be included in the study, participants must report the value given for N in the story. Any participant who gives the wrong answer for any values of N is completely excluded from the study.

Context: Tour Boat, Everyone Acts			
	Fishermen's Utility	John's Utility	Frustrated Means
Better	0.099	0.624	0.802
Same	0.040	0.257	0.099
Worse	0.861	0.119	0.099

Context: Tour Boat, Only John Acts			
	Fishermen's Utility	John's Utility	Frustrated Means
Better	0.082	0.888	0.918
Same	0.786	0.071	0.041
Worse	0.133	0.041	0.041

Context: Fisherman, Everyone Acts			
	Fishermen's Utility	John's Utility	Frustrated Means
Better	0.185	0.326	0.533
Same	0.120	0.152	0.087
Worse	0.696	0.522	0.380

Context: Fisherman, Only John Acts			
	Fishermen's Utility	John's Utility	Frustrated Means
Better	0.074	0.884	0.926
Same	0.726	0.053	0.042
Worse	0.200	0.063	0.032

Table S1. Responses from subjects in the Expected Utility group in Study 3. Cells contain the percentage of subjects that responded better, same, or worse to each of the three questions (Fishermen's Utility, John's utility, and Frustrated Means). See above for the exact wording of each question.

2. How many people regularly fish in Lake Wilson in the summer? To be included, subjects must answer 19, 20, or 21. Any participant who gives the wrong answer for any values of N is completely excluded from the study.

3. How many people, besides John, are actually going to use the new hooks? To be included, subjects must answer 0. Any participant who gives the wrong answer for any values of N is completely excluded from the study.

4. Will it make a difference to the fish population if John uses the new hooks? There are three possible answers to this question: (A) It will make the fish population more healthy (B) It will make the fish population less healthy (C) It will not make a difference to the fish population. To be included, subjects must answer (C). Any participant who gives the wrong answer for any values of N is completely excluded from the study.

Exclusions: 350 subjects were excluded from the study for answering 1 or more of the check questions incorrectly in one or more of the conditions.

Subjects read the following instructions prior to beginning the study: On the following pages you will be asked to read a short story and answer a few pages of questions about it. The story will remain largely the same from page to page, but after every few pages of questions, the story will change slightly. When the story changes, we will **bold and color** the part of the story that is different, so you can see the difference easily. After the survey there will be an opportunity to let us know if something was confusing or unclear.

Subjects read the following stimuli. On each new page, the story appeared again for subjects' reference. Presented below is the 4,7 Condition. The 10,13 Condition was identical except that the numbers 10 and 13 replace the numbers 4 and 7 (respectively) in the third paragraph.

–Page 1–

Lake Wilson is a small lake in upstate New York. Each summer, a few dozen families move into small cottages near the lake for the season. The vacationers enjoy boating, swimming, and fishing in the lake and they've gotten to know each other over the course of many summers together.

Most people who like to fish in Lake Wilson catch a few fish each day and cook them for dinner. These people buy their fishing gear from a small tackle shop on the main road. Up until now, the tackle shop has sold one kind of fishing hook. But it has recently starting selling a new kind of hook that allows people to catch many more fish than they would with the older hook.

There are 20 people who regularly fish in Lake Wilson during the summer. If 7 or more people started using the new hook for fishing, then by the end of the summer, the entire fish population would collapse and there would be no more fish in Lake Wilson ever again. None of the vacationers would want that to happen. However, up to 4 people can use the new hooks with no decrease at all in the size and health of the fish population. Those people will be able to catch more fish more quickly, everyone else will still be able to catch the same amount of fish as before, and the fish population would continue to be exactly as large and as healthy as in past years.

It is impossible to tell what hook someone is using from a distance, so if someone starts using the new hooks, no one else will know about it. No one has bought the new hooks yet, however. John would like to use the new hooks because he would like to catch more fish faster. He is thinking about whether or not to buy the new hooks.

John wants to find out what his neighbors think about the new hooks, so he decides he is going to visit the cottages of all the people who fish in Lake Wilson and ask them. John manages to talk to the other 19 people who regularly fish in the lake. When John sits down with each person to talk about the new hooks, this is what he finds out:

N people say something like this: "I would love to use those new hooks to catch more fish faster, but what would happen if everyone did that? If everyone used the new hooks, all the fish would disappear from the lake. I don't want that to happen, so I'm not going to use the new hook."

On the other hand, N people say: "I'm just not interested in using those new hooks. I really only need to catch a few fish a day, and I like to do that a leisurely pace and spend all day fishing."

John thinks to himself: No one else is going to use the new hooks. Under these circumstances, it's OK for me to use the new hooks and catch more fish.

Please answer the following comprehension questions, so we know that you read the story.

If John starts using the new hooks, how many people will know about it? [Free Response.]

How many people, besides John, would like to use the new hooks if there were no bad effects of doing so? [Free Response.]

How many people regularly fish in Lake Wilson in the summer? [Free Response.]

How many people, besides John, are actually going to use the new hooks? [Free Response.]

–Page 2–

Is it morally acceptable for John to use the new hooks? [Yes/No]

–Page 3–

Is there a rule on the lake that the new hooks are forbidden? [Yes/No]

–Page 4–

Will it make a difference to the fish population if John uses the new hooks? [It will make the fish population more healthy/It will make the fish population less healthy/It will not make a difference to the fish population]

Subjects repeated this process until all the values of N were seen. The color of the bolded text was changed for each new value of N.

Supplemental Results/Statistical Details. A linear regression shows that there was no significant impact of the number of interested parties on judgments of how many people would know that John was using the new hook ($F = 0.39, t = -0.63, p = 0.53$). Moreover, when knowledge judgments are entered into a regression with interested parties (as well as the interaction), knowledge is not a significant predictor of moral judgments ($z = -0.14, p = 0.89$) nor is the knowledge $\times$ interested parties interaction ($z = 0.79; p = 0.43$).

Study 4b

Materials. This study was pre-registered (see http://aspredicted.org/blind.php?x=at7cs8). 300 subjects participated in this study, recruited from Amazon MTURK through turkprime and were paid a small amount for their participation. Subjects were randomly assigned to one of two conditions (4,7 Condition and 10,13 Condition) and one of three collective action questions (yielding a 2×3 design). 4,7 Condition: Up to 4 people can use the new hook with no effect on the fish population; once 7 people use the new hook the fish population will go extinct. 10,13 Condition: Up to 10 people can use the new hook with no effect on the fish population; once 13 people use the new hook the fish population will go extinct. The three collective action questions were Everyone's Expected Utility, John's Expected Utility, and Frustrated Means (described below).

Each subject read the story and was asked what would happen if N subjects used the new hook (exact wording varied depending on the collective action curve, see below). N was chosen at random without replacement from the following values until all values of N were seen by each subject: 0,2,7,8,13,19. [This list is for John's EU and Frustrated Means. For Fishermen's EU, subjects see N+1.]

Pre-Registered Exclusion Criteria:

How many people regularly fish in Lake Wilson in the summer? To be included, subjects must answer 20. Any participant who gives the wrong answer for any values of N is completely excluded from the study.

Exclusions: 18 subjects were excluded from the experiment for failing the control question.

Subjects read the following instructions prior to beginning the study: "On the following pages you will be asked to read a short story and answer some questions about it. After the survey there will be an opportunity to let us know if something was confusing or unclear."

Below, we show the stimuli for the 4,7 Condition. The 10,13 condition is identical, except that the numbers 4 and 7 were replaced by the numbers 10 and 13 (respectively) in the third paragraph.

Lake Wilson is a small lake in upstate New York. Each summer, a few dozen families move into small cottages near the lake for the season. The vacationers enjoy boating, swimming, and fishing in the lake and they've gotten to know each other over the course of many summers together.

Most people who like to fish in Lake Wilson catch a few fish each day and cook them for dinner. These people buy their fishing gear from a small tackle shop on the main road. Up until now, the tackle shop has sold one kind of fishing hook. But it has recently starting selling a new kind of hook that allows people to catch many more fish than they would with the older hook.

There are 20 people who regularly fish in Lake Wilson during the summer. If 7 or more people started using the new hook for fishing, then by the end of the summer, the entire fish population would collapse and there would be no more fish in Lake Wilson ever again. None of the vacationers would want that to happen. However, up to 4 people can use the new hooks with no decrease at all in the size and health of the fish population. Those people will be able to catch more fish more quickly, everyone else will still be able to catch the same amount of fish as before, and the fish population would continue to be exactly as large and as healthy as in past years.

The last two sentences of the story varied by collective action curve question:

Fishermen's Expected Utility: Think about the well-being of all the vacationers combined. How would the vacationers be affected if the following number of people used the new hooks?

John's Expected Utility: John is one of the people who visits Lake Wilson each summer. John would like to use the new hooks. How would John be affected if the following number of people also used the new hooks?

Frustrated Means: John is one of the people who visits Lake Wilson each summer. He would like to use the new hooks to catch more fish than he did last year using the old hooks. Some of John's neighbors are also thinking about using the new hooks.

Subjects were then asked the following questions, depending on the collective action curve question:

Fishermen's Expected Utility: Imagine that N people use the new hooks. How would the vacationers be affected? [N is replaced with the values indicated above.] Subjects give a response ranging from -50 to 50 with the scale anchored as follows: A lot worse off, a little worse off, not affected, a little better off, a lot better off.

John's Expected Utility: Imagine that John and N other people use the new hooks. How would John be affected? [N is replaced with the values indicated above.] Subjects give a response ranging from -50 to 50 with the scale anchored as follows: A lot worse off, a little worse off, not affected, a little better off, a lot better off.

Frustrated Means: Imagine that John and N other people use the new hooks. How likely is it that John will be able to catch more fish than he did last year? [N is replaced with the values indicated above.] Subjects give a response ranging from -50 to 50 with the scale anchored as follows: very unlikely, somewhat unlikely, neither likely nor unlikely, somewhat likely, very likely.

At the end, all subjects were asked the following question: How many people regularly fish in Lake Wilson in the summer? [Free Response.]

Supplemental Results/Statistical Details. We next describe our model fitting and model comparison procedure for Study 4b.

Model fit using empirical utility function: Using Equation 1 from the main text (our model of universalization)

$$Prob(\text{Acceptable; Universalization}) = \frac{1}{1 + e^{\tau(U(0)-U(n_i))+\beta}} \qquad [1]$$

we assigned a probability to each moral judgment collected in Study 4a, restricting our analysis to those participants whose moral judgments were not uniform across all values of n_i (the number of interested parties). Equation [1] specifies these probabilities given $U(0)-U(n_i)$ and two free parameters τ and β. In Study 4b we collected an empirical measure of $U(0)-U(n_i)$ for each value of n_i required to model moral judgment data from Study 4a (i.e., 1, 3, 8, 9, 14, and 20) crossed with each of the relevant utility threshold conditions (i.e., 4-7 or 10-13). We computed the mean of $U(0) - U(n_i)$ across Study 4b participants for each of these points, and substituted those means into Equation [1]. Using the "optim" function in R, we selected values of τ and β which, when applied uniformly across all moral judgments and all participants, maximized the likelihood of the moral judgment data given our model. We applied $\tau = 2$ and $\beta =$ -3 as starting points for the optimization procedure, and we obtained the optimized values $\tau = 0.057$ and $\beta =$ -1.16. The minimized sum of the negative log likelihood was $nll = 302.3$ and the AIC was calculated as $2p + 2nll = 608.8$ where $p = 2$ is the number of free parameters.

Model fit using idealized utility function: We next performed the same procedure but substituted idealized values of $U(0) - U(n_i)$ in place of the empirically derived ones described above. The idealized utility function was defined as having a flat positive utility before the critical threshold and a flat negative utility after the threshold. The precise model predictions within the "threshold region" (i.e., between 4 and 7 in the 4,7 Condition and between 10 and 13 in the 10,13 Condition) are unimportant because we did not gather subject moral judgments in that range, so there is no data to predict. Therefore, any of the possible ideal models described in the main text (see the introduction to Study 4) would make nearly identical predictions. We used a step-function model that is flat before the threshold and after the threshold and fit it to the data using a 2-parameter sigmoid, analogous to Equation [1]. The threshold for the 4,7 condition was set at 4 and for the 10,13 condition was set at 10. Thus, for $n_i <$ threshold, $U(0) - U(n_i) = 1$ and for $n_i >$ threshold, $U(0) - U(n_i) = 0$. We fit parameters as above except with starting points $\tau = 0$ and $\beta = 0$ and resulting optimized values $\tau = 2.37$ and $\beta = 0.847$. The minimized sum of the natural log likelihood was $nll = 309$ and the AIC was calculated as $2p + 2nll = 621$ where $p = 2$.

In the main text, we include only the data from the Fishermen's Expected Utility Condition. Here, we report the data for the other two conditions. The empirical utility functions produced by the John's Expected Utility and Frustrated Means Conditions can be found in Figs. S9 and S10 respectively.

Comparing alternative measures of $U(n)$ As an alternative method of modeling the data without restricting the analysis to non-uniform responders, we also conducted the following pre-registered analysis of all the collective action data. The purpose of this analysis was to compare the likelihood of the data from Study 4a given our universalization model when applying each of the three different utility measures collected in Study 4b: Fishermen's EU, John's EU and Frustrated Means.

As above, empirical utility functions were created by taking the average subject response at each number of parties acting (1, 3, 8, 9, 14, 20) for each threshold condition (4,7 or 10,13). We determined the likelihood of the data given a variant of Equation [1] that differs in two key respects. First, it eliminates the bias parameter β. Second, it imposes empirically-derived upper and lower bounds on $Prob$(Acceptable) corresponding to the proportion of participants who judged John's action impermissible even when $n_i = 0$ and the proportion of participants who participants who judged John's action permissible even when $n_i = 20$. In other words, the model bounds accommodate participants who tend to provide uniform moral judgments, applying the logistic function just within the range of participants whose judgments are plausible candidates for universalization. For the 10,13 condition, max = .80 and min = .58. For the 4,7 condition, max = .80 and min = .60. Thus, our modified model was

$$Prob(\text{Acceptable}) = \frac{\max - \min}{1 + e^{-\tau(U(0)-U(n_i))}} + \min.$$

As above, we used the optim function of R to find the value of τ that maximized the probability of the data given our model. We did this separately for each measure of $U(0) - U(n_i)$ collected in Study 4b.

We compared the model fits using their AIC (see Table S2). Individual AIC values are difficult to interpret, so we re-scale AIC to

$$\Delta_i = AIC_i - AIC_{min}$$

where AIC_min is the minimum AIC value of the set of models to be compared. Therefore, Δ_i can be interpreted as the information loss of using a model other than the best-fitting one and allows for a strength-of-evidence comparison and the generation of a ranked list of the models (2). The convention for comparing Δ_i values is generally taken to be as follows: models with $\Delta_i \leq 2$ are considered to have substantial support, models with $4 \geq \Delta_i \geq 7$ are taken to have less support and models with $\Delta_i > 10$ are taken to have almost no support .

Fishermen's EU has the lowest AIC of the three models for both thresholds. For the 4,7 threshold, John's EU has substantial support when compared to the best model while Frustrated Means has less support. For the 10,13 threshold, John's EU and Frustrated Means both have less support than the best model. This suggests that across the two conditions, there is somewhat greater support for the model that uses Fishermen's EU to predict moral judgments.

However, none of the models were clearly and consistently superior to all the others across both thresholds (see (2)). For this reason, we conducted Study 3 (see main text) which differentiates between the models.

**Threshold**		**Fishermen's Expected Utility**	**John's Expected Utility**	**Frustrated Means**
**4,7**	Starting point for parameter optimization	0.40	2.00	0.05
	Optimized Beta	0.24	2.36	0.047
	Sum of NLL	653.9	655.1	658.2
	AIC	1310	1312	1318
	Δ_i	0	2	8
**10,13**	Starting point for parameter optimization	0.40	0.10	0.05
	Optimized Beta	0.32	2.36	0.067
	Sum of NLL	604.2	606.1	606.7
	AIC	1210	1214	1215
	Δ_i	0	4	5

Table S2. Details for optimization of model fits for Study 4b data. These models predict the moral acceptability data reported in Study 4a.

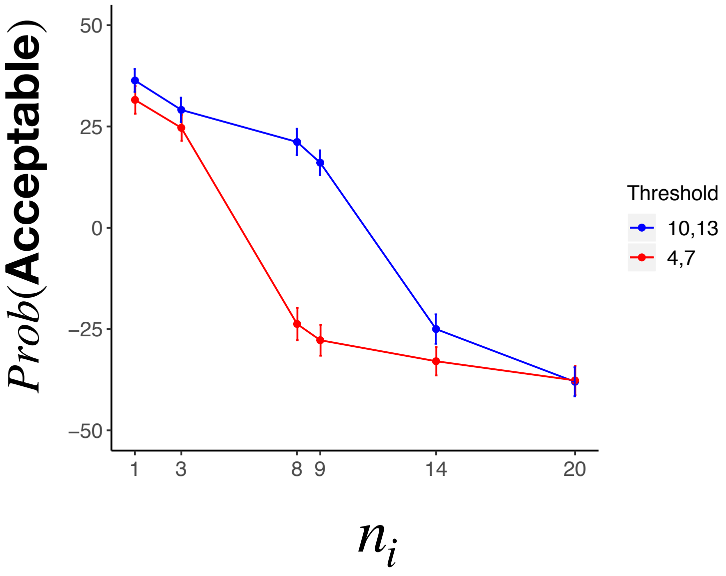

Fig. S9. Empirical utility function produced by asking subjects about John's Expected Utility.

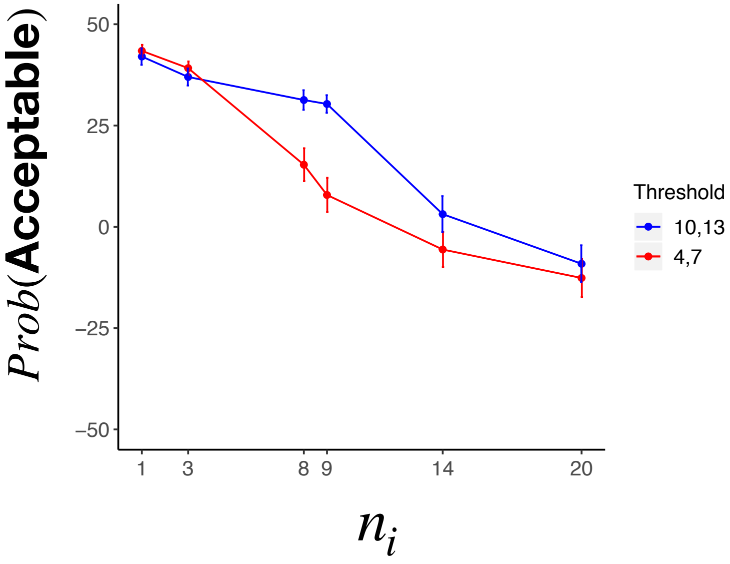

Fig. S10. Curve produced by asking subjects about the likelihood that John's purpose would be undermined.

Study 5

Materials. 4-11 year old children were recruited for participation in the Boston Common. Parents gave informed consent for their children to participate. Testing took place in a quiet location outdoors in the Common. Testing sessions were video recorded and checked in case there was a question about a subject's response. Responses were coded online by a live coder. Subjects were given a small prize for participating.

191 subjects were included in the analysis (mean age = 7.5 years). 28 additional children were recruited but excluded from the analysis for failing the screening or control questions.

Children were first told simple stories accompanied by pictures to verify their competence with English and to ensure that they could use "OK" and "not OK" to make simple moral judgments. Subjects were not corrected if they got any of the screening questions wrong.

Pictures and animations used for this study are available at github.com/sydneylevine/universalization.

Story 1: This is a story about Billy and Johnny. In this story, Billy hits Johnny.

Was that OK or not OK? [Correct Answer: Not OK]

Story 2: This is a story about and Anne. What is Sue holding? That's right, a flower! In this story, Sue gives her flower to Anne.

Was that OK or not OK? [Correct Answer: OK]

Story 3: Sometimes Jimmy is all alone and sometimes he's with his friends. When he is all alone sometimes he does things that are OK and sometimes he does things that are not OK.

One day Jimmy is all alone. There is no one else around, he is all by himself. He knows that if he steps on this bunny, no one will see him do it.

Q: If Jimmy steps on the bunny, will anyone see him do it? [Correct Answer: No]

DV: Is it OK or not OK for Jimmy to step on the bunny? [Correct Answer: Not OK]

Story 4: One day Jimmy is all alone. There is no one else around, he is all by himself. He knows that if he makes a picture for his mom, no one will see him do it.

Q: If Jimmy makes a picture for his mom, will anyone see him do it? [Correct Answer: No]

DV: Is it OK or not OK for Jimmy to make a picture for him mom? [Correct Answer: OK]

If subjects got any of the screening stories wrong, the study ended after the screening. For subjects that passed the screening, they went on to the main study. Subjects listened to two stories, counterbalancing condition (High Interest or Low Interest) and Context (Buckets or Rocks). They were asked a series of control questions during the story. If a child got a control question wrong, the experimenter repeated that part of the story again and asked the question again. If the child still got the question wrong, he or she was excluded from the study. At the end of the story the subject was asked if it would be OK or not OK for the actor to do the action (e.g., take a rock from the path). They were then asked why they made the judgment that they did. Analysis of justifications is not presented in this paper.

Condition: High interest

Context: Buckets

This is a story about Mary. Mary and her friends are at the lake. The fish in the lake love swimming in the lake water.

Mary has a toy castle. And she has a bucket. All the other kids have toy castles too and they all have buckets.

Mary would like to fill a bucket with water from the lake and pour it on her toy castle. All the other kids want to fill their buckets with water and pour it on their castles.

Q: What does Mary want to do?

Q: Do the other kids want to do that?

If just one person takes water from the lake there will still be plenty of water left for the fish. If everyone takes water from the lake, then there would be no more water in the lake for the fish.

Q: What would happen if just one person took water from the lake?

If don't know, follow up with: Would there still be water left for the fish?

Q: What would happen if everyone took water from the lake?

If don't know, follow up with: Would there still be water left for the fish?

The kids don't want that to happen. So they never take water from the lake.

Q: Do the kids ever take water from the lake?

One day, Mary is at the lake before any of the other kids. She knows that if she fills her bucket with water and pours it on her castle, no one will see her do it. And she'll do it just this once.

Q: If Mary takes water from the lake, will anyone see her do it?

So, I have a question for you about Mary, are you ready?

DV: Is it OK or not OK for Mary to take water from the lake?

Explain: Why do you think it would be OK/not-OK?

Condition: Low interest

Context: Buckets

This is a story about Mary. Mary and her friends are at the lake. The fish in the lake love swimming in the lake water.

Mary has a toy castle. And she has a bucket. All the other kids have toy castles too.

Mary would like to fill a bucket with water from the lake and pour it on her toy castle. If just one person takes water from the lake there will still be plenty of water left for the fish. If everyone takes water from the lake, then there would be no more water in the lake for the fish.

Q: What would happen if just one person took water from the lake?

If don't know, follow up with: Would there still be water left for the fish?

Q: What would happen if everyone took water from the lake?

If don't know, follow up with: Would there still be water left for the fish?

But no one else wants to take water from the lake. They don't want their castles to be wet; they like them to stay nice and dry. They never take water from the lake because they don't want the water on their castles. Only Mary wants to do that.

Q: What does Mary want to do?

Q: Do the other kids want to do that?

One day, Mary is at the lake before any of the other kids. She knows that if she fills her bucket with water and pours it on her castle, no one will see her do it. And she'll do it just this once.

Q: If Mary takes water from the lake, will anyone see her do it?

So, I have a question for you about Mary, are you ready?

DV: Is it OK or not OK for Mary to take water from the lake?

Explain: Why do you think it would be OK/not-OK?

Condition: High interest

Context: Rocks

This is a story about Jacob. Jacob and his friends walk through this park every day. They love to walk on this path that is made of lots of rocks.

Jacob has a rock collection. He would love to take one of the rocks from the path and put it in his rock collection. All the other kids have rock collections, too. They would all love to take the rocks from the path and put them in their rock collections.

Q: What does Jacob want to do?

Q: Do the other kids want to do that?

If just one person took a rock for his rock collection, there would still be plenty of rocks left on the path. If everyone took the rocks for their rock collections, then there would be no more rocks left on the path.

Q: What would happen if just one person took a rock for his rock collection?

If don't know, follow up with: Would there still be rocks left on the path?

Q: What would happen if everyone took the rocks for their rock collections?

If don't know, follow up with: Would there still be rocks left on the path?

The kids don't want that to happen. So they never take the rocks.

Q: Do the kids ever take the rocks from the path?

One day, Jacob is at the park before any of the other kids. He knows that if he takes a rock from the path, no one will see him do it. And he'll do it just this once.

Q: If Jacob takes a rock from the path, will anyone see him do it?

So, I have a question for you about Jacob, are you ready?

DV: Is it OK or not OK for Jacob to take a rock from the path?

Explain: Why do you think it would be OK/not-OK?

Condition: Low interest

Context: Rocks

This is a story about Jacob. Jacob and his friends walk through this park every day. They love to walk on this path that is made of lots of rocks.

Jacob has a rock collection. He would love to take one of the rocks from the path and put it in his rock collection. If just one person took a rock for his rock collection, there would still be plenty of rocks left on the path. If everyone took the rocks for their rock collections, then there would be no more rocks left on the path.

Q: What would happen if just one person took a rock for his rock collection?

If don't know, follow up with: Would there still be rocks left on the path?

Q: What would happen if everyone took the rocks for their rock collections?

If don't know, follow up with: Would there still be rocks left on the path?

But none of the other kids want to take rocks from the path. They don't like rocks that much and they don't have rock collections. So the other kids never take rocks from the path. Only Jacob wants to do that.

Q: What does Jacob want to do?

Q: Do the other kids want to do that?

One day, Jacob is at the park before any of the other kids. He knows that if he takes a rock from the path, no one will see him do it. And he'll do it just this once.

Q: If Jacob takes a rock from the path, will anyone see him do it?

So, I have a question for you about Jacob, are you ready?

DV: Is it OK or not OK for Jacob to take a rock from the path?

Explain: Why do you think it would be OK/not-OK?

H. Adults. 201 adult subjects received the same stimuli as children, except they did not see the screening stories. Adults were recruited from Amazon MTURK through turkprime and were paid a small amount for their participation. Thirty-three subjects were excluded for failing control questions. Like children, adults saw two stories, counterbalanced for condition (High Interest or Low Interest) and context (Buckets or Rocks). The data from both stories is analyzed.

Supplemental Results/Statistical Details. We planned to analyze the data from children using a Bayesian analysis, which avoids the need for a pre-determined sample size (3–6), due to our uncertainty about the effect size for this study and the difficulty of recruiting subjects. In the results section, we report the Bayes Factor as the main item of analysis, though we also include p-values to conform with current standards for data reporting.

To test for an effect of age, we compared three models of the data. Model-1 includes condition only, Model-2 includes the main effect of age, Model-3 includes an age $\times$ condition interaction. In the latter two models, there is no significant effect of age or the age $\times$ condition interaction and the data is best explained by the model that includes only condition on AIC and BIC. See Fig. S12. Fig. S11 shows subject responses by age.

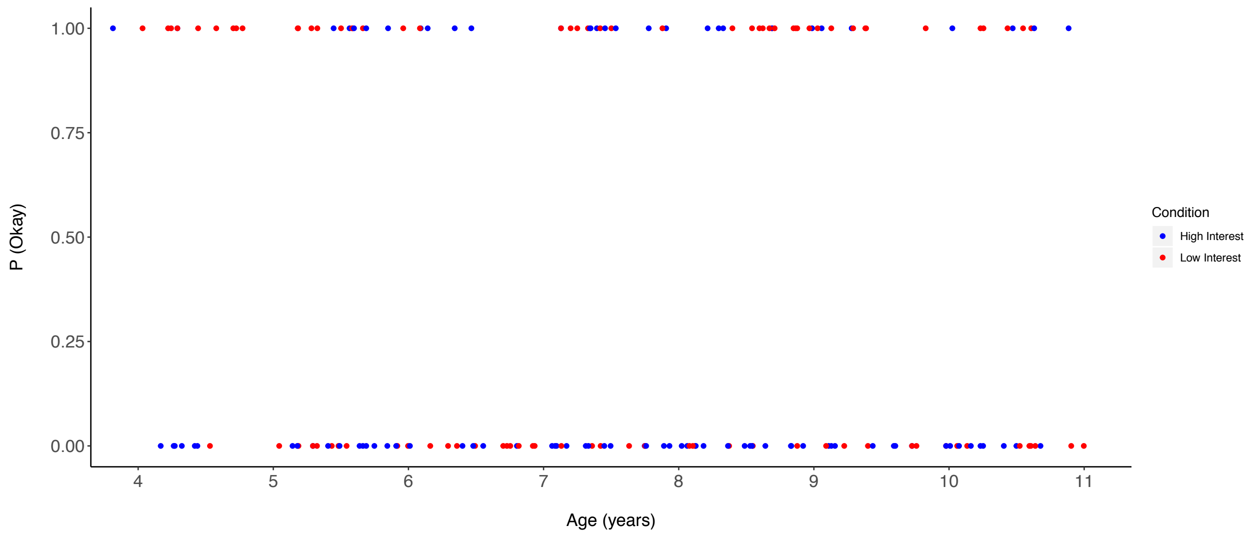

Fig. S11. Scatter plot of children's responses by age and condition. Only answers to the first story are graphed.

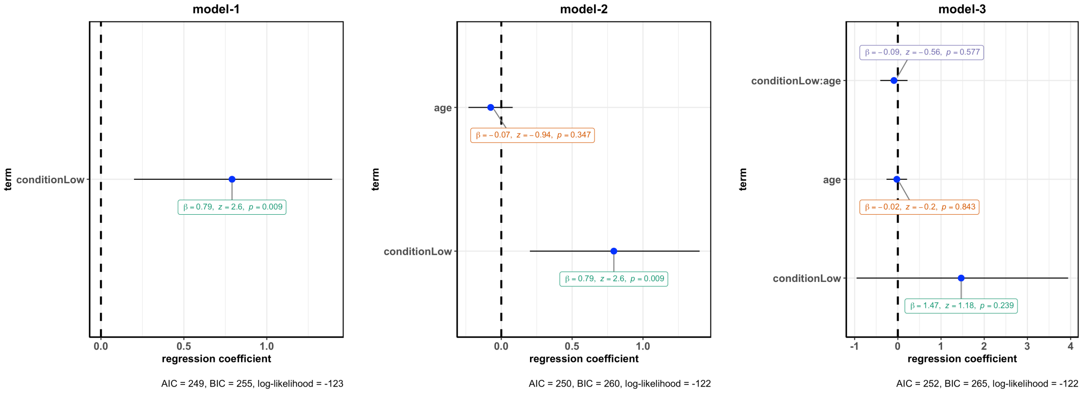

Fig. S12. We compared three models of the data. Model-1 includes condition only, Model-2 includes the main effect of age, Model-3 includes an age $\times$ condition interaction. In the latter two models, there is no significant effect of age or the age $\times$ condition interaction and the data is best explained by the model that includes only condition on AIC and BIC.

References

1. I Patil, ggstatsplot: 'ggplot2' based plots with statistical details. *CRAN* (2018).
2. KP Burnham, DR Anderson, Multimodel inference: understanding aic and bic in model selection. *Sociol. methods & research* **33**, 261–304 (2004).
3. JN Rouder, Optional stopping: No problem for bayesians. *Psychon. bulletin & review* **21**, 301–308 (2014).
4. EJ Wagenmakers, M Lee, T Lodewyckx, GJ Iverson, Bayesian versus frequentist inference in *Bayesian evaluation of informative hypotheses.* (Springer), pp. 181–207 (2008).
5. EJ Wagenmakers, A practical solution to the pervasive problems ofp values. *Psychon. bulletin & review* **14**, 779–804 (2007).
6. W Edwards, H Lindman, LJ Savage, Bayesian statistical inference for psychological research. *Psychol. review* **70**, 193 (1963).
